# Genipin Attenuates Diabetic Cognitive Impairment by Reducing Lipid Accumulation and Promoting Mitochondrial Fusion via FABP4/Mfn1 Signaling in Microglia

**DOI:** 10.3390/antiox12010074

**Published:** 2022-12-29

**Authors:** Wanying Liu, Ke Li, Menglin Zheng, Ling He, Tong Chen

**Affiliations:** Department of Pharmacology, China Pharmaceutical University, 639, Longmian Avenue, Nanjing 211198, China

**Keywords:** diabetic cognitive impairment, genipin, microglia, FABP4, Mfn1

## Abstract

The present study was conducted to evaluate the effect of genipin (GEN) on the microglia of diabetic cognitive impairment and explore its potential mechanism. Diabetic mice were induced by STZ/HFD, while GEN was intragastrically and intraventricularly treated. The human microglia cell HMC3 was induced by LPS/HG/PA. As a result, GEN attenuated diabetic symptoms and diabetic cognitive impairment-related behavior in novel object recognition, Morris water maze and passive avoidance tests. GEN inhibited M1 microglia polarization, lipid accumulation, oxidative stress and promoted mitochondrial fusion via FABP4/Mfn1. FABP4 overexpression, Mfn1 overexpression, selective FABP4 inhibitor BMS, and Mfn1 SiRNA were employed for investigating the mechanism. The inhibitory effect of GEN on ROS may be associated with NOX2 signaling and the translocation of p47phox/p67phox to the cell membrane. With the ROS scavenger NAC, it was proved that ROS participated in GEN-mediated inflammation and lipid accumulation. GEN inhibited the phosphorylation and nucleus translocation of NF-κB. GEN inhibited the ubiquitination of Mfn1, which was mediated by the E3 ligase Hrd1. GEN also enhanced microglia phagocytosis. Molecular docking predicted that GEN may interact with FABP4 by hydrogen bond at the S53 and R78 residues. In conclusion, GEN attenuated diabetic cognitive impairment by inhibiting inflammation, lipid accumulation and promoting mitochondrial fusion via FABP4/Mfn1 signaling.

## 1. Introduction

Diabetes mellitus is a chronic metabolic disease endangering the health and life quality of patients around the world. The incidence rate of diabetes mellitus has been growing in recent decades. As the major form of diabetes, type 2 diabetes mellitus (T2DM) induces various complications in the heart, kidney, foot, eye and central nervous system. Diabetic encephalopathy is a common complication of diabetes, encompassing neurobehavioral deficiency, cognitive impairment and learning/memory disability. Diabetic cognitive impairment is characterized by insulin resistance, hyperglycemia, hyperlipidemia, mitochondria dysfunction, decreased hippocampal synaptic plasticity, excited microglia, oxidative stress and inflammatory reaction. T2DM patients are more likely to suffer from cognitive dysfunction than T1DM patients. With 50% more risk of dementia, T2DM is related to impaired attention, executive function and verbal memory. Type 1 diabetes mellitus (T1DM) patients show various cognitive deficits associated with visuospatial ability, motor speed and psychomotor efficiency. A high-fat diet (HFD) is classically used to mimic obesity, dyslipidemia, insulin resistance and reduced sugar tolerance in diabetes. Streptozotocin (STZ) is the pancreas β islet cytotoxic antibiotic relying on its nitrosourea group. The single injection of high-dosage STZ (almost 180 mg/kg) is commonly used to induce T1DM, whereas the injection of less than 65 mg/kg of STZ for two or three times combined with HFD is usually applied for T2DM murine model. The etiology of diabetic cognitive disorders remains elusive [1,2,3]. The patients with diabetic cognitive impairment are usually treated by anti-diabetic drugs, but specific drugs are still warranted. Therefore, it is urgent to discover the pathogenesis of diabetic cognitive impairment and develop its potential drugs.

Neuroinflammation is acknowledged to initiate and exacerbate cognitive impairment. The uncontrolled activation of microglia contributes to the secretion of large amounts of inflammatory cytokines in the brain, which is implicated in the lesion of the neuro-immune system. Microglia are globally viewed as the tissue macrophage in the central nervous system. Microglia secret inflammatory cytokines and clear debris in response to central inflammation. Various stimuli including chemokine, cytokine, endotoxin and hyperglycaemia activate microglia. Microglia have been defined as different types of polarization, including classical (M1) type and alternative (M2) type activation. M1 phenotype microglia are featured by tumor necrosis factor (TNF)-α, Interleukin (IL)-6, inducible nitric oxide synthase (iNOS), C-C Motif Chemokine Ligand 2 (CCL2) and CD68, while IL-4, IL-10, Arginase 1 (Arg1), Chitinase-like protein 3 (Ym1) and CD206 are the hallmarks of M2 phenotype microglia. The balance of M1 phenotype microglia and M2 phenotype microglia is crucial for the immune homeostasis of the central nervous system [4,5]. The activation of M1 microglia has been found in the brains of db/db mice [6]. The inhibition of M1 phenotype microglia and the induction of M2 phenotype microglia are commonly believed to relieve neuroinflammation and diabetic encephalopathy [7]. However, the mechanism of microglia activation in diabetic cognitive impairment has not been fully elucidated.

Fatty acid (FA) is a member of the carboxylic acids and plays a crucial role in biological progression. FA content is positively related to multiple metabolic syndromes, including T2DM. The fatty acid-binding protein (FABP) is one of the prerequisites for FA trafficking by increasing the water solubility of FA. FABP4, also known as A-FABP, is a lipid chaperone expressed in adipocytes, endothelial cells and macrophages [8,9]. Previous evidence displayed that FABP4 mediated lipid-associated metabolism in obesity, type 2 diabetes mellitus, schizophrenia and cognition [10,11]. The insulin resistance and obesity were notably inhibited in FABP4 knockout mice [12]. The suppression of FABP4 was reported to relieve the symptom of diabetes and diabetic encephalopathy [6]. FABP4 was positively correlated with TNF-α and IL-6 in the blood of gestational diabetes mellitus patients [13]. FABP4 ablation also attenuated neuroinflammation in microglia by reliving mitochondria dysfunction and inhibiting iNOS and TNF-α expressions [14]. Thus, we assumed that FABP4 may be a promising target for the intervention of diabetic cognitive impairment. BMS309403 (BMS) is the selective FABP4 inhibitor, which has been investigated both in vivo and in vitro. The roles of FABP4 in diabetic cognitive impairment and microglia have not yet been fully understood.

Upon the alterations of immune and metabolic reaction, mitochondria are able to modify their morphological shape via fission and fusion, namely mitochondrial dynamics. In response to high glucose, excessive fatty acid and inflammatory storm, mitochondria elongate to achieve lower density matrix and more cristae. This fission process is governed by dynamin-related protein 1 (Drp1). Fusion allows for mitochondria to compensate for functional deficiency by sharing proteins and ribosomal RNAs. Excessive fatty acid influences mitochondrial fusion proteins mitofusin (Mfn) 1 and 2. Mfn1 and Mfn2 drive the fusion of the outer membrane of mitochondria (OMM), which initiates the fusion progression [15]. The shift toward mitochondrial fusion is generally acknowledged to improve energy metabolism and prevent reactive oxygen species (ROS) overproduction [16]. The weakened antioxidant defense system accelerates mitochondrial dysregulation including mitochondrial fission and altered mitochondrial membrane potential via uncontrolled ROS [17]. The effect of mitochondrial fusion in microglia, especially the microglia in hyperglycemia/hyperlipidemia environments, has not yet been well defined.

Genipin (GEN), a bioactive compound extracted from the gardenia fruit of Gardenia jasminoides Ellis, has been reported to exhibit anti-diabetic, anti-oxidative and anti-inflammatory activities. GEN was commonly elicited to treat diabetes and its complications [18,19]. It was discovered that GEN relieved neuroinflammation in CUMS-induced depression [20]. Although GEN has been proposed to inhibit inflammatory reaction in microglia, the mechanism by which GEN modulates microglia polarization remains not fully understood [21]. Moreover, the effect of GEN on diabetic cognitive impairment, fatty acid accumulation or mitochondrial fusion in the central nervous system has been scarcely reported. The present research was carried out to investigate the pharmacological effect of GEN on diabetic cognitive impairment via fatty acid accumulation and mitochondrial fusion in microglia. The mechanism by which GEN mediated FABP4 and Mfn1, as well as the stress of microglia in high glucose/high fatty acid stimulation, were researched. Moreover, the relationship between FABP4-mediated fatty acid accumulation and Mfn1-mediated mitochondrial fusion was explored. The mediation of Mfn1 expression including ubiquitination and its E3 ligase was also investigated.

## 2. Materials and Methods

### 2.1. Reagents

Genipin (GEN, with purity over 98%) was purchased from Kailai (Xi’an, China). BMS-309403 (BMS), cycloheximide (CHX), MG132, and acetylcysteine (NAC) were produced by MedChemExpress (Shanghai, China). Metformin hydrochloride (MET) was purchased from Jiangsu Suzhong Pharmaceutical Group (Taizhou, China). The drugs were dissolved in DMSO and normal saline [(the concentration of DMSO was less than 0.1% (*w*/*v*)). Lipopolysaccharide (LPS, L2880) and STZ (s0130) were supplied from Sigma-Aldrich (St. Louis, MO, USA). Bodipy 493/503 (D3922) was provided by Thermo Fisher (Shanghai, China). Palmitic acid (PA), supplied from Aladdin (Shanghai, China), was dissolved in fatty acid-free albumin from bovine serum (BSA) (ST025, Beyotime, Nanjing, China) and ethanol. FITC-Dextran was supplied from Santa Cruz Biotechnology (sc-263323, Santa Cruz, CA, USA) and Sigma-Aldrich (St. Louis, MO, USA).

Iba1 (MA527726), CD206 (12-2069-42), Mfn1 (MA5-24789, PA5-117691), p-Drp1-S637 (PA5-101038) and ubiquitin (13-1600) were purchased from Thermo Fisher (Shanghai, China). FABP4 (ab92501), CD68 (ab201340), NADPH oxidase (NOX)2 (ab129068), p67phox (ab175293) and Hrd1 (ab170901) antibodies were obtained from Abcam (Cambridge, UK). Mfn2 (#9482S), Drp1 (#8570S), p-Nuclear Factor Kappa B (NF-κB) (#3033), NF-κB (#8242), CD68 (#91882), p47phox (#4312), β-actin (#4970), Na, K-ATPase (#3010), ubiquitin (#3936), Flag (#8146), Flag (#14793), HA (#3724), Myc (#2276), V5 (#80076), mouse anti-rabbit IgG (Conformation Specific, #5127), rabbit anti-mouse IgG (Light Chain Specific, #58802) and mouse anti-rabbit IgG (Light-Chain Specific, #93702) antibodies were provided by Cell Signaling Technology (Danvers, MA, USA). HA (m180-3) and Myc (562-5) antibodies were supplied from MBL (Beijing, China). CD68 (25747-1-AP) was provided by Proteintech (Wuhan, China). Iba1 (sc-32725) and Mfn1 (sc-166644) antibodies were obtained from Santa Cruz Biotechnology (Santa Cruz, CA, USA).

### 2.2. Animals

Male ICR mice (18 g–22 g) were purchased from Qinglongshan Animal Culture Farm 5 days prior to the experiment. The animals were housed in standard lab with 12 h/12 h day/night cycles at 25 ± 1 °C. The mice had free access to food and water. All experiments were conducted according to the National Institutes of Health Guidelines. All efforts were achieved to reduce the sacrifice and suffering of the animals.

### 2.3. Diabetic Model and Treatment

The mice were assigned to a sham group (n = 15) and a model group. The animals in the model group were fed with HFD including 77% regular diet, 15% lard, 5% white sugar, 2% cholesterol, 0.7% salt and 0.3% sodium cholate. The mice were intraperitoneally injected with 60 mg/kg streptozotocin (STZ), which was dissolved in sodium citrate–hydrochloric acid buffer (pH 4.5) for three times at the sixth week. The glucometer and glucose test strips (Accu-Check Aviva, Roches, Basel, Switzerland) were employed to assess the blood glucose levels. Only the mice with blood glucose over 15 mmol/L were used for the following test. The diabetic mice were randomly divided into STZ/HFD group, STZ/HFD + metformin (MET, 200 mg/kg) group, STZ/HFD + GEN (10 mg/kg) group, STZ/HFD + GEN (20 mg/kg) group and STZ/HFD + GEN (40 mg/kg) group. MET served as the positive control. From the 7th week, the animals were intragastrically treated with GEN four times a week for 4 weeks. The mice in the STZ/HFD + GEN (10, 20, 40 mg/kg) group were also treated with GEN (2.5, 5, 10 μg/mouse, respectively) by brain stereotaxic injection once at the 7th and 9th weeks. Oral glucose tolerance test (OGTT) and insulin tolerance test (ITT) were conducted at the 10th week. Then, the behavior tests including novel object recognition, Morris water maze and passive avoidance tests were conducted to estimate the cognitive impairment from the 11th week.

### 2.4. Brain Stereotaxic Injection

After the anesthesia by pentobarbital, the mice were fixed in a stereotaxic equipment (Taimeng, Chengdu, China). The skin was cut along the sagittal suture and subcutaneous tissue was carefully separated. In order to wipe the skull mucosa, 30% hydrogen peroxide was used. The bregma was regarded as the origin of coordinate system and two holes (0.3 mm posterior, ± 1.0 mm lateral and 3.0 mm ventral from bregma) were drilled. GEN (2.5, 5, 10 μg/mouse) was injected for 5 min. The needle retention was conducted for 1 min to avoid leakage. The skins were disinfected and sutured. Finally, the mice were warmed on an electric blanket. The mice in the sham group suffered the same surgical operation and were stereotaxically injected with vehicle at the same time.

### 2.5. Behavior Tests

#### 2.5.1. Novel Object Recognition Test

The novel object recognition test was employed to estimate the memory and recognition of diabetic mice. The mouse was separately placed facing the wall in plastic apparatus (50 cm × 50 cm × 50 cm) with two black plastic Objects A (5 cm × 5 cm × 5 cm) 20 cm apart. The adaptation trail lasted for 5 min. After 24 h, one Object A was replaced with a green cylinder Object B (diameter: 3 cm, height: 6 cm). The mouse was still individually allowed to explore freely in the chamber for 5 min. The recognition was identified when the mouse touched or sniffed the object or when the distance between the object and the nose was smaller than 0.2 cm. In order to clear the odor during the interval of two single trails, 75% ethanol was used. The discrimination time was monitored. The discrimination index (DI) was defined as (novel object exploration time—familiar object exploration time)/(total exploration time).

#### 2.5.2. Morris Water Maze (MWM) Test

The spatial memory and learning ability were evaluated by MWM, which consisted of 4-day orientation navigation tests and a probe trial on the fifth day. The MWM test was conducted using a circular pool (120 cm diameter, 50 cm height) with 30 cm depth 25 ± 1 °C water and a 9 cm diameter platform. The platform with a flag was visible in one quadrant on days 1–2 and was hidden at 1 cm below water without the flag on days 3–4. Each mouse was individually placed facing the wall and allowed to swim for 90 s. The mice were allowed to rest for 15 s no matter whether they arrived or did not arrive at the platform. In the probe trial, the platform and flag were both removed. The animals had 90 s to search the platform. The platform crossings, the time spent in the target quadrant and the escape latency to achieve the platform were recorded.

#### 2.5.3. Passive Avoidance Test

The passive avoidance apparatus contains a lit compartment and a darkened compartment with a grid floor and a guillotine gate. In the acquisition trial, each mouse was electrically shocked (0.2 mA, 3 s) when it entered the dark room. Thus, the mouse returned to the lit room. After 24 h, the mouse was placed again in the illuminated compartment and allowed to walk freely for 5 min during the probe trail. The escape latency and the error number for the entries of the dark room were recorded.

### 2.6. OGTT and ITT

The mice were fasted overnight and then their blood glucose was assessed (0 min). For OGTT, the mice intragastrically received 1.8 g/kg glucose. For ITT, the mice were intraperitoneally treated with 0.75 U/kg insulin. Afterward, the blood glucose was recorded at 30, 60 and 120 min. Then, the area under concentration-time curve (AUC) in OGTT and ITT were calculated.

### 2.7. Cell Culture, Treatment and Transfection

Human microglial cells HMC3 and 293T were provided by ATCC. The cells were cultured with Dulbecco’s Modified Eagle’s Medium (BC-M-005, Biochannel, Nanjing, China) complemented with 10% fetal bovine serum (BC-SE-FBS01, Biochannel, Nanjing, China), penicillin (100 U/mL) and streptomycin (100 µg/mL) (C100C5, Xinsaimei, Soochow, China). The cells were incubated in an incubator with 5% carbon dioxide at 37 °C. The cells were passaged upon 80% confluence. The cell passage was carried out every 2–3 days. Only the cells in the logarithmic growth phase were used in the present research.

Following this, 4 × 10^4^ cells/mL were seeded onto a 96-well plate or a 6-well plate for 24 h. The cells were treated with GEN (5 μM, 10 μM, 20 μM), BMS (40 μM) or NAC (1 mM) for 4 h and then stimulated with LPS (1 μg/mL), high glucose (HG, 33 mM) and PA (100 μM) for another 12 h.

HMC3 cells were transfected with 5′-Cholesteroi SiRNA for Mfn1 which was provided by Genepharma (Shanghai, China). The SiRNA and Lipofectamine 2000 or Lipofectamine 3000 (11668019, L3000001, Invitrogen, Carlsbad, CA, USA) were mixed with opti-MEM (Gibco, Carlsbad, CA, USA) for 5 min. Then, two mixtures were blended for 20 min prior to the incubation with cells, or directly added to the cells. After 4 h, the culture medium was renewed for another 48 h of incubation. After the transfection, the cells were treated with GEN (5 μM, 10 μM, 20 μM) or BMS (40 μM) for 4 h and then stimulated with LPS (1 μg/mL), high glucose (HG, 33 mM) and PA (100 μM) for another 12 h.

Homo 3× Myc-Mfn1, 3× Flag-Ub, HA-Hrd1 or V5-FABP4 plasmids were provided by Genewiz (Soochow, Jiangsu, China). The plasmids were amplificated and their DNA concentrations were assessed by Nano-300 (Allsheng, Hangzhou, Zhejiang, China). The transfection was carried out using Lipofectamine 2000 or Lipofectamine 3000 and opti-MEM as the above description. After the transfection, the cells were treated with GEN (20 μM) or BMS (40 μM) for 4 h and then stimulated with LPS (1 μg/mL), high glucose (HG, 33 mM) and PA (100 μM) for another 12 h.

### 2.8. ELISA

The hippocampal tissues were rapidly removed after the sacrifice of the mice. The tissues were gently washed by ice PBS, weighted and washed. Then, the samples were diluted at the ratio of 1 g tissue:9 mL PBS. The tissue and cell samples were homogenized with RIPA (P0013, Beyotime, Nanjing, China) on ice. After the centrifugation at 3000 rpm, the supernatant was collected. The protein concentration was calculated using BCA commercial kit (P0012, Beyotime, Nanjing, China). The hippocampal and supernatant contents of TNF-α, IL-6, IL-4 and IL-10 were determined in accordance with the ELISA kits (Elabscience, Wuhan, China).

### 2.9. Flow Cytometry

For the measurement of ROS, the cells were collected, centrifugated at 1500 rpm for 5 min and resuspended. After the incubation with DCFH-DA (final concentration of 10 μM diluted by serum-free culture medium) at 37 °C for 30 min, the cells were centrifugated at 1000 rpm for 5 min and washed by PBS. The fluorescence reactivity was visualized by flow cytometry (Beckman Coulter, Brea, CA, USA) with 500 nm excitation wavelength and 525 nm emission wavelength.

For the measurement of CD68 and CD206, the cells were centrifugated at 1500 rpm for 5 min and resuspended with PBS. The cells were incubated with CD68 and CD206 antibodies on a shaking table for 30 min. The antibodies were diluted in PBS as follows: CD206: 5 µL (0.125 µg)/test. CD68: 0.5 µL (0.20 µg)/test. Then, the cells were washed and filtered prior to the detection using flow cytometry. The results were analyzed by flowjo.

For the measurement of FITC-Dextran, the cells were centrifugated at 1500 rpm for 5 min and resuspended with PBS. The cells were incubated for 30 min with 5 µL FITC-Dextran dissolved in 30 µL PBS containing 2% fetal bovine serum. Then, the cells were washed and filtered prior to the detection using flow cytometry. The results were analyzed by flowjo.

### 2.10. Oxidative Stress Indicators

The levels of malondialdehyde (MDA) (A003-1-2, A003-2-2), superoxide dismutase (SOD) (A001-3-2), glutathione peroxidase (GSH-Px) (A005-1-2) and the ratio of nicotinamide adenine dinucleotide phosphate (NADP^+^)/Reduced Nicotinamide Adenine Dinucleotide Phosphate (NADPH) (A115-1-1) were measured as recommended in the instrument of the kits (Jiancheng, Nanjing, China). For MDA, the cells were prepared to 10% homogenate using PBS and the absorbance was monitored at 532 nm. For SOD, the cells were diluted at the ratio of 10^6^ cells to 0.4 mL PBS and the absorbance was recorded at 450 nm. For GSH-Px, the cells were prepared to 10% homogenate using PBS and the absorbance was measured at 412 nm. For NADP^+^/NADPH, the cells were diluted to acid extract or alkaline extract at the ratio of 5 × 10^7^ cells to 1 mL. The absorbance was detected at 570 nm. The protein concentrations were calculated by BCA kit (P0012, Beyotime, Nanjing, China).

### 2.11. Total Cholesterol (TC) and Triacylglycerol (TG)

The levels of TC and TG in serum were examined by commercial kits (A111-1-1, A110-1-1, Jiancheng, Nanjing, China) in accordance with the instrument.

### 2.12. Oil Red O Staining

About 1 × 10^5^ cells were washed by PBS twice and fixed by 4% paraformaldehyde. Oil red O staining was conducted using the commercial kit (C0158S, Beyotime, Nanjing, China). The cells were exposed to 1 mL dyeing detergent for 20 s, and then oil red O staining solution for 30 min. Thereafter, the cells were washed by 1 mL dyeing detergent for 30 min and stained by hematoxylin for 10 s. The lipid accumulation was observed under a microscope.

### 2.13. JC1

JC1 commercial kit (M8650, Solarbio, Beijing, China) was applied for the visualization of mitochondrial membrane potential. A 50 μL JC1 (200×) solution was diluted with 8 mL ultrapure water, to which was added with 2 mL JC1 dyeing buffer (5×) to obtain JC1 working solution. Following this, 2 × 10^4^ cells were gently washed with 1 mL culture medium and treated with 1 mL JC1 working solution for 20 min in incubator. Then, 1 mL JC1 dyeing buffer (5×) was added with 4 mL distilled water to obtain JC1 dyeing buffer (1×). Then, the cells were exposed to iced JC1 dyeing buffer (1×). Afterward, the supernatant was discarded and replaced with 2 mL culture medium. The mitochondrial membrane potential was observed immediately under fluorescence microscope (Cytation 5, BioTek, Beijing, China).

### 2.14. Transmission Electron Microscopy (TEM)

The hippocampi were isolated rapidly on ice after the sacrifice of mice and fixed in electron microscope specific fixative solution (G1102, Servicebio, Wuhan, China). The cells were also exposed to this electron microscope specific fixative solution and gently scraped. All the samples were transported in 4 °C, wrapped in 1% agarose and then fixed with 1% osmic acid for 2 h. Afterward, the samples were dehydrated by graded ethanol for 20 min each and exposed to 100% acetone twice for 15 min. The infiltration embedment was carried out by acetone and 812 embedding agent (1:1, 37 °C, 8 h). The samples were placed at 37 °C overnight and at 60 °C for 48 h. The resin block was sliced using Leica UC7 and then stained by 2% uranium acetate for 8 min in dark. The section was washed with 70% ethanol 3 times, 2.6% lead citrate for 8 min without carbon dioxide and finally observed under TEM (HT7800/HT7700, HITACHI).

### 2.15. Polymerase Chain Reaction (PCR)

After the homogenization using Trizol, the total RNA of the hippocampal tissues and cells were extracted with Trizol and isopropanol. RNA was washed with 75% ethanol and dissolved by DEPC. The RNA concentration was determined by Nano-300 micro-spectrophotometer (Allsheng, Hangzhou, China). The reverse transcription was carried out using a HiScript^®^ III All-in-one RT SuperMix Perfect for qPCR kit (R333, Vazyme, Nanjing, China) according to the manufacturer’s instruction. The reaction system contained 4 μL 5 × All-in-one qRT SuperMix, 1 μL Enzyme Mix and 1 μg template RNA. The reverse transcription was conducted at 50 °C for 15 min and at 85 °C for 15 s. Taq Pro Universal SYBR qPCR Master Mix (Q712, Vazyme, Nanjing, China) was used for amplification. The mixture contained 10 μL 2 × Taq Pro Universal SYBR qPCR Master Mix, 0.4 μL forward primer (10 μM) and 0.4 μL reverse primer (10 μM). PCR was conducted at 95 °C for 3 min, 95 °C for 10 s and 60 °C for 30 s (40 cycles). Finally, it was conducted at 95 °C for 15 s, 60 °C for 60 s and 95 °C for 15 s. The mRNA expressions were normalized to β-actin and presented as relative gene expression by 2^−ΔΔCt^ equation. The primer sequence is illustrated in Table 1.

### 2.16. Immunofluorescence Staining

The mice were sacrificed and cardiac perfused with 4% paraformaldehyde. The brain tissues were fixed by 4% paraformaldehyde before frozen section. The cells were fixed in 4% paraformaldehyde for 30 min. The samples were permeabilized with 0.3% Triton X-100 and blocked by 5% BSA for 1.5 h. Then, the slides or cells were incubated with primary antibodies at 4 °C overnight. After washing, the samples were incubated by secondary antibodies including IgG H&L (Alexa Fluor^®^ 488) (ab150077), IgG H&L (Alexa Fluor^®^ 555) (ab150114) or IgG H&L (Alexa Fluor^®^ 594) (ab150116) at room temperature for 1 h in a dark environment. The samples were washed with PBS, stained by DAPI (10 μL/mL) and then sealed by anti-fluorescence quenching sealing solution (P0126, Beyotime, Nanjing, China) in the dark. The immunofluorescence observation was conducted under confocal microscope (LSM700, Carl Zeiss, Oberkochen, BW, German) or fluorescent microscope (BioTek Cytation 5).

The cells were treated with mitotracker (M22426, Thermo Fisher, Shanghai, China) (C1049B or C1048, Beyotime, Nanjing, China) at 37 °C for 30 min. MitoSOX (M36008, Thermo Fisher, Shanghai, China) was applied for the detection of mitochondrial ROS. The cells were washed by HBSS (G4204, servicebio, Nanjing, China). The immunofluorescent was evaluated using a confocal microscope (LSM700, Carl Zeiss).

### 2.17. Western Blot

The hippocampal tissues and cells were homogenized with RIPA lysis buffer on ice for 30 min. The cell lysates were centrifuged at 4 °C at 12,000 rpm and the protein concentration was determined using BCA commercial kit (P0012, Beyotime, Nanjing, China). The membrane protein was isolated by the standard kit (P0033, Beyotime, Nanjing, China). Then, the protein was heated with loading buffer at 95 °C for 5 min. A total of 40 μg protein was subjected to 8–15% SDS-polyacrylamide gel and transferred onto the polyvinylidene fluoride membrane. The membrane was blocked by 5% skim milk at room temperature and incubated with primary antibodies at 4 °C overnight. The dilution ratios were as follows: FABP4 (1:1000), Mfn1 (1:1000), p-NF-κB (1:1000), NF-κB (1:1000), Mfn2 (1:1000), p-Drp1-S637 (1:1000), Drp1 (1:1000), β-actin (1:1000), NOX2 (1:5000), p47phox (1:1000), p67phox (1:1000) and Na, K-ATPase (1:1000). Thereafter, the membrane was washed and incubated with a horseradish peroxidase (HRP)-conjugated secondary antibody. The immuno-intensity was visualized with an enhanced chemiluminescence system (Tannon, Nanjing, China).

### 2.18. Coimmunoprecipitation (Co-IP)

After the transfection and treatment, the cells were washed with PBS and lysed using iced IP buffer containing 150 mM NaCl, 1 mM EDTA, 1 mM EGTA, 50 nM Tris, 1% Trition X-100, 2 mM DTT, 100 μM PMSF and 1 μg/mL Proteinase for 30 min. The lysates were centrifugated at 4 °C for 20 min at 12,000 rpm. The protein concentration of the supernatant was calculated by BCA kit. A/G magnetic beads (B23202, Bimake, Shanghai, China) were precleared and resuspended with IP buffer without proteinase, PMSF and DTT. Afterward, the samples were incubated with a primary antibody at 4 °C overnight. The IP antibody dilution ratio of Mfn1 was 1:200 and the dilution ratio of 3× Myc was 1:250. After the incubation with A/G magnetic beads again at 4 °C for another 2 h, the samples were boiled with loading buffer and applied for Western blot detection. The IB antibody dilution ratios of ubiquitin and 3× Flag were all 1:1000.

### 2.19. Molecular Docking

Molecular docking was carried out using Discovery Studio 2019 with FABP4 receptor (PDB: 5D4A) and GEN (chemical book: 6902-77-8). The protein and compound were prepared. Then, the binding site between receptor and ligand was defined according to the endogenous ligand. After CDOCKER performance, the hydrogen bonds were analyzed.

### 2.20. Statistical Analysis

The experimental results in the present research were described as means ± SDs. Significance was analyzed by one-way analysis of variance (ANOVA) followed by Tukey’s multiple comparisons test or two-way ANOVA with Bonferroni’s post hoc analysis using the GraphPad Prism 7.0 and 9.4 software. *p* < 0.05 was considered as statistically significant.

## 3. Results

### 3.1. GEN Inhibited Microglia Inflammation and Lipid Accumulation in LPS/HG/PA-Induced HMC3 Cells

**Figure 1:**
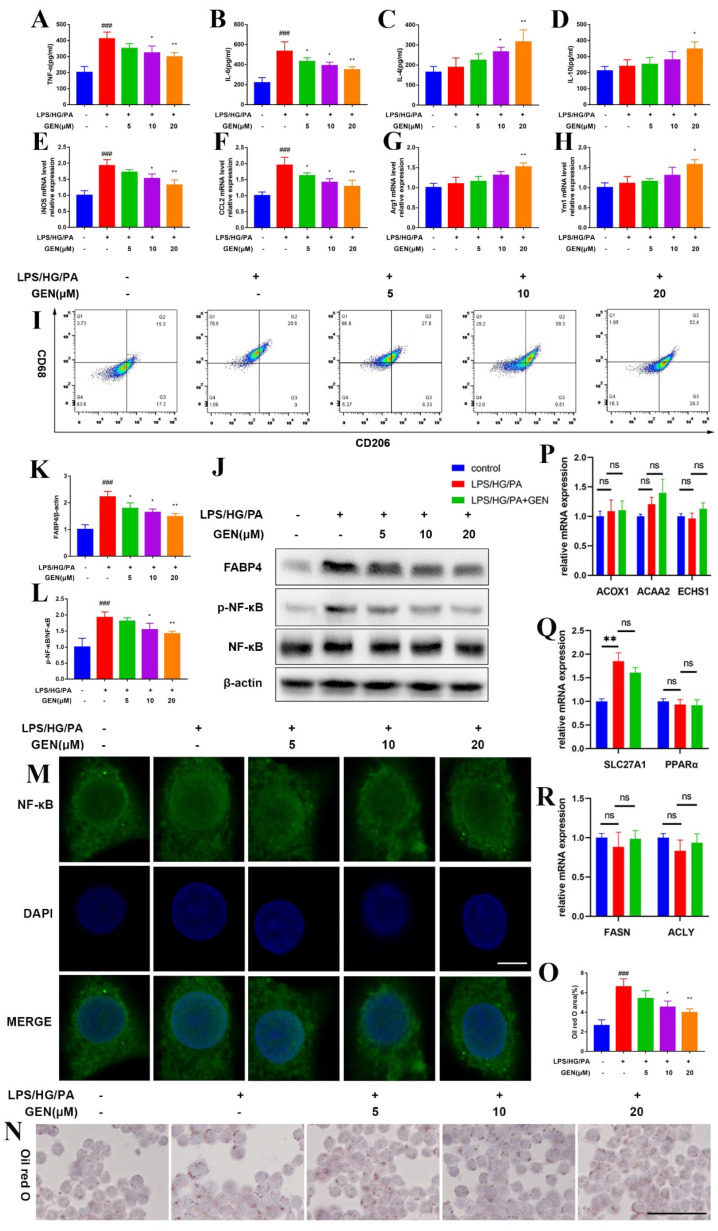
GEN shifted microglia polarization and inhibited lipid accumulation in LPS/HG/PA-induced HMC3 cells. The HMC3 cells were treated with GEN (5, 10, 20 μM) for 4 h and then stimulated with LPS/HG/PA for 12 h. The supernatant concentrations of TNF-α (**A**), IL-6 (**B**), IL-4 (**C**) and IL-10 (**D**) were examined by ELISA (n = 4). The mRNA expressions of *iNOS* (**E**), *CCL2* (**F**), *ARG1* (**G**) and *YM1* (**H**) in cells were assessed by PCR (n = 4). The population of CD68-positive and CD206-positive cells were measured by flow cytometry (**I**). The expressions of FABP4, p-NF-κB and NF-κB were detected by Western blot (**J**–**L**) (n = 3). The nucleus translocation of NF-κB was visualized by immunofluorescence staining under laser confocal microscope. The scale bar equaled 5 μm (**M**). The lipid accumulation was observed by oil red O staining. The scale bar equaled 50 μm (**N**). The analysis of oil red O staining (**O**). The mRNA expressions of fatty acid β-oxidation genes including *ACOX1*, *ACAA2* and *ECHS1* were measured by PCR (**P**). The mRNA expressions of fatty acid uptake genes including *SLC27A1* and *PPARα* were measured by PCR (**Q**). The mRNA expressions of fatty acid synthesis genes including *FASN* and *ACLY* were measured by PCR (**R**). The results are expressed as means ± SDs. ^###^ *p* < 0.001 compared with control group. * *p* < 0.05, ** *p* < 0.01 compared with LPS/HG/PA group or the other group. ns means not significant.

We further detected several protein expressions of the samples in our previous research with another bioactive compound extracted from gardenia fruit of Gardenia jasminoides Ellis, and found that GEN relieved lipid accumulation, mitochondrial dynamics and inflammation [22]. As microglia are crucial cells participating in the inflammation/immune response, we hoped to investigate the underlying mechanism. Polarization is the major inflammatory modulated format of microglia; thus, we estimated the effect of GEN on microglia polarization in response to LPS/HG/PA stimulation. GEN (5, 10, 20 μM) treatment remarkably reduced the expressions of M1 phenotype biomarkers TNF-α, IL-6, *iNOS* mRNA and *CCL2* mRNA in LPS/HG/PA-induced HMC3 cells (Figure 1A,B,E,F). GEN (5, 10, 20 μM) treatment also augmented the expressions of the M2 phenotype biomarkers IL-4, IL-10, *ARG1* mRNA and *YM1* mRNA (Figure 1C,D,G,H). The expressions of CD68 and CD206 were detected by flow cytometry. GEN inhibited CD68 expression and elevated CD206 expression in LPS/HG/PA-induced HMC3 cells (Figure 1I). It was noteworthy that GEN exhibited a stronger regulatory property on M1 microglia polarization than M2 microglia polarization. Therefore, LPS/HG/PA stimulation caused inflammatory response, while GEN treatment exhibited anti-inflammatory property. As the essential inflammatory mediator, NF-κB is activated and then translocated into the nucleus to promote the transcription of inflammatory cytokines in response to inflammation, hyperglycemia and hyperlipidemia. We verified the effect of GEN on NF-κB by immunofluorescence. As expected, GEN prevented the nucleus translocation of NF-κB (Figure 1M). Lipid staining was conducted using the oil red O method. GEN was found to inhibit lipid accumulation (1N-O). Next, we evaluated the mRNA expressions of fatty acid β-oxidation genes, fatty acid uptake genes and fatty acid synthesis genes. The fatty acid β-oxidation genes including Acyl-CoA Oxidase 1 (*ACOX1*), Acetyl-CoA Acyltransferase 2 (*ACAA2*), Enoyl-CoA Hydratase, Short Chain 1 (*ECHS1*) (Figure 1P) and fatty acid synthesis genes including Fatty Acid Synthase (*FASN*) and ATP Citrate Lyase (*ACLY*) (Figure 1R) were not significantly altered by LPS/HG/PA stimulation nor GEN (20 μM) treatment. The mRNA expressions of fatty acid uptake genes including Solute Carrier Family 27 Member 1 (*SLC27A1*) and Peroxisome Proliferator Activated Receptor α (*PPARα*) were also examined. LPS/HG/PA stimulation notably increased *SLC27A1* mRNA while GEN treatment did not significantly reduce *SLC27A1* transcription (Figure 1Q). Thus, we further assessed another fatty acid uptake family of FABPs, especially those expressed in the brain, namely *FABP3*, *FABP4*, *FABP5* and *FABP7*. It was found that the LPS/HG/PA challenge prominently upregulated *FABP4* and *FABP5* transcriptions in HMC3 cells, whereas GEN (20 μM) treatment downregulated *FABP4* mRNA expression (Figure S1). The protein expression of FABP4 and NF-κB phosphorylation were detected by Western blot. GEN treatment inhibited the protein levels of FABP4 and p-NF-κB (Figure 1J–L). Our data implied that GEN inhibited inflammation and lipid accumulation via FABP4/NF-κB signaling in LPS/HG/PA-induced HMC3 cells.

### 3.2. GEN Promoted Mitochondrial Fusion and Reduced Oxidative Stress in LPS/HG/PA-Induced HMC3 Cells

**Figure 2:**
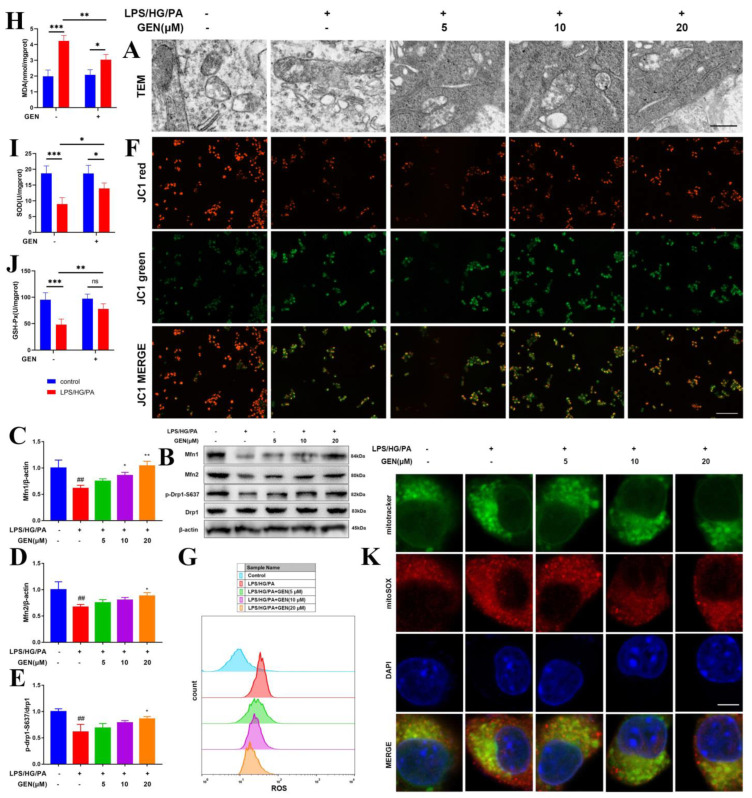
GEN promoted mitochondrial fusion and inhibited oxidative stress in LPS/HG/PA-induced HMC3 cells. The HMC3 cells were treated with GEN (5, 10, 20 μM) for 4 h and then stimulated with LPS/HG/PA for 12 h. The mitochondrial morphology was observed by TEM. The scale bar equaled 500 nm (**A**). The protein expressions of Mfn1, Mfn2, p-S637-Drp1 and Drp1 were detected by Western blot (**B**–**E**) (n = 3). The mitochondrial membrane potential was visualized by JC1. The scale bar equaled 100 μm (**F**). ROS-positive cell counts were evaluated by flow cytometry. The x axis represented dichlorofluorescein (DCF), an ROS indicator. The y axis represented cell count (**G**). GEN (20 μM) ameliorated MDA, SOD and GSH-Px (**H**–**J**) (n = 4). The mitoSOX and mitotracker were observed by immunofluorescence staining. The scale bar equaled 5 μm (**K**). The results are expressed as means ± SDs. ^##^ *p* < 0.01 compared with control group. * *p* < 0.05, ** *p* < 0.01, *** *p* < 0.001 compared with LPS/HG/PA group or the other group. ns means not significant.

The mitochondrion is a crucial organelle in inflammation, diabetes, obesity and cognitive deficiency. We observed the morphological change of mitochondria using TEM. As shown in Figure 2A, the LPS/HG/PA exposure caused mitochondrial fission and elongation, which was prevented by GEN treatment. The incubation with GEN also contributed to mitochondrial fusion (Figure 2A). The protein expressions of mitochondrial fusion indicators including Mfn1, Mfn2 and p-S637-Drp1 were detected. We found that GEN augmented the expressions of these fusion proteins, especially Mfn1 (Figure 2B–E). Mitochondrial membrane potential was visualized by JC1. LPS/HG/PA stimulation reduced mitochondrial membrane potential, which was reversed by GEN treatment (Figure 2F). As mitochondria was critical for oxidative stress, we estimated the levels of MDA, SOD, GSH-Px and ROS. GEN treatment reduced the content of MDA, reduced ROS positive cell counts and increased the activities of SOD and GSH-Px (Figure 2G–J). The mitochondrial ROS was observed by mitoSOX and mitotracker. GEN treatment decreased the fluorescence intensity of mitoSOX (Figure 2K). Our results display that GEN treatment promoted mitochondrial fusion and decreased oxidative stress in LPS/HG/PA-induced HMC3 cells.

### 3.3. GEN Attenuated Oxidative Stress by Inhibiting NOX2 Signaling and the Translocation of p47phox/p67phox to Cell Membrane

**Figure 3:**
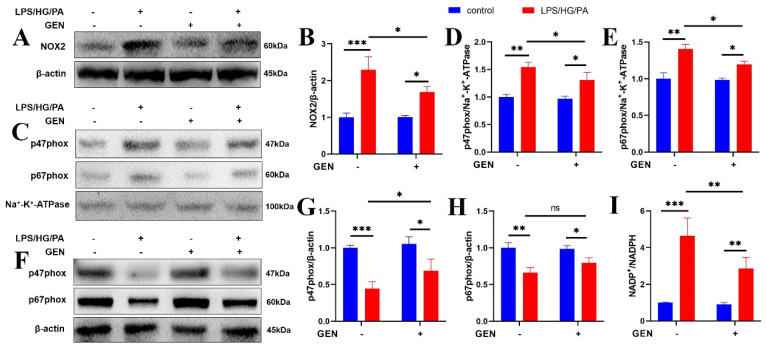
GEN relieved oxidative stress by inhibiting NOX2 and the translocation of p47phox/p67phox to the cell membrane. The HMC3 cells were treated with GEN (20 μM) for 4 h and then stimulated with LPS/HG/PA for 12 h. The protein expression of NOX2 was detected by Western blot (**A**,**B**). The protein expressions of p47phox and p67phox in cell membrane were detected by Western blot (**C**–**E**). The protein expressions of p47phox and p67phox in cytoplasm were detected by Western blot (**F**–**H**) (n = 3). The NADP^+^/NADPH ratio was detected (**I**) (n = 4). The results were expressed as means ± SDs. * *p* < 0.05, ** *p* < 0.01, *** *p* < 0.001 compared with the other group. ns means not significant.

NOX2 signaling was the classical event for ROS generation in microglia. However, the effects of GEN on NOX2 signaling were scarcely reported. It was found that GEN inhibited NOX2 expression (Figure 3A,B). GEN also inhibited the translocation of p47phox and p67phox into the cell membrane (Figure 3C–H). GEN treatment reduced the NADP^+^/NADPH ratio (Figure 3I). The ROS scavenger NAC was used for further investigation. It was found that without ROS, the attenuated effects of GEN on TNF-α and IL-6 were abrogated (Figure S2A,B). NAC also hampered the inhibitory effect of GEN on lipid accumulation (Figure S2C,D). The data demonstrated that GEN attenuated oxidative stress by inhibiting NOX2 signaling and the translocation of p47phox/p67phox to the cell membrane. ROS was required for GEN-ameliorated inflammation and lipid accumulation.

### 3.4. FABP4 Inhibition or Mfn1 Overexpression Inhibited Inflammation and Lipid Accumulation in LPS/HG/PA-Induced HMC3 Cells

To further investigate the mechanism, the FABP4 selective inhibitor BMS and the Mfn1 overexpression plasmid were used. As depicted in Figure S3A,B, BMS evidently reduced the concentrations of TNF-α and IL-6. The protein expressions of FABP4 and p-NF-κB were inhibited, and Mfn1 was upregulated by BMS (Figure S3C–F). The lipid accumulation was suppressed by BMS treatment (Figure S3G,H). BMS also reduced the ROS positive cell population (Figure S3I). The data indicated that the inhibition of FABP4 by BMS reduced inflammatory cytokines, suppressed lipid accumulation, restrained oxidative stress and promoted mitochondrial fusion in LPS/HG/PA-induced HMC3 cells.

The Mfn1 overexpression efficiency was identified in Figure S4C. The transfection with Mfn1 plasmid decreased the concentrations of TNF-α and IL-6 (Figure S4A,B). Mfn1 plasmid prevented the phosphorylation of NF-κB (Figure S4D–F). The immunofluorescence staining with Mfn1 and bodipy showed that Mfn1 overexpression reduced bodipy intensity. Moreover, Mfn1 and bodipy exhibited co-location (Figure S4G). The results demonstrate that Mfn1 overexpression inhibited inflammation and lipid accumulation in LPS/HG/PA-induced HMC3 cells.

### 3.5. Mfn1 Was Required for the Ameliorated Effect of GEN and BMS on Inflammation and Lipid Accumulation

**Figure 4:**
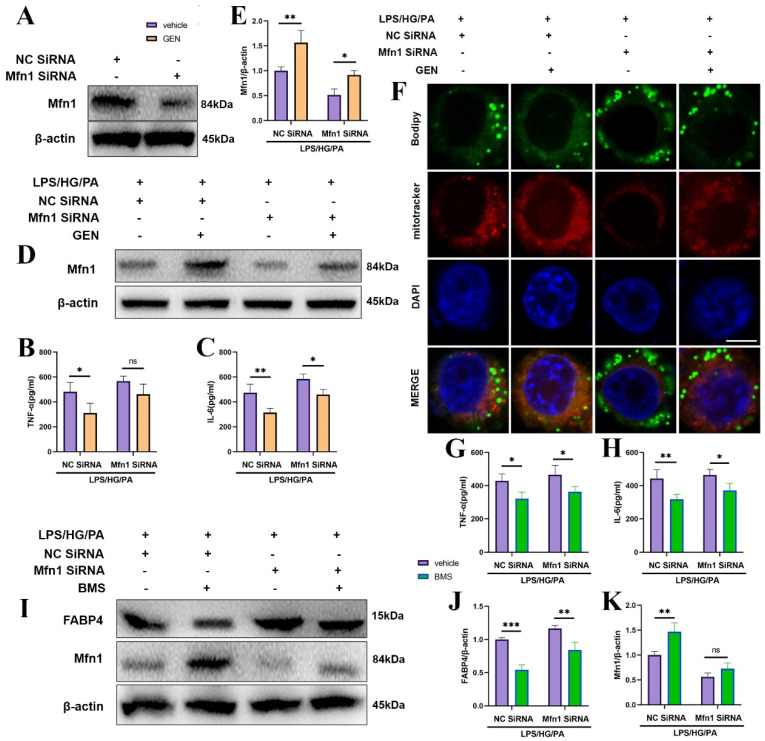
The effects of GEN or BMS on inflammation and lipid accumulation were abrogated by Mfn1 SiRNA. The HMC3 cells were transfected with Mfn1SiRNA or NC SiRNA prior to the treatment with GEN (20 μM) for 4 h. Then, the cells were challenged by LPS/HG/PA for 12 h. The knockdown efficacy was verified by Western blot (**A**) (n = 3). The supernatant concentrations of TNF-α and IL-6 were examined by ELISA (**B**,**C**) (n = 4). The Mfn1 protein expression was detected by Western blot (**D**,**E**) (n = 3). The bodipy and mitotracker were observed by immunofluorescence staining under laser confocal microscope. The scale bar equaled 5 μm (**F**). The HMC3 cells were transfected with Mfn1 SiRNA or NC SiRNA prior to the treatment with BMS for 4 h. Then, the cells were challenged by LPS/HG/PA for 12 h. The supernatant concentrations of TNF-α and IL-6 were examined by ELISA (**G**,**H**) (n = 4). The protein expressions of FABP4, Mfn1 were detected by Western blot (**I**–**K**) (n = 3). The results were expressed as means ± SDs. * *p* < 0.05, ** *p* < 0.01, *** *p* < 0.001 compared with the other group. ns means not significant.

Next, the roles of Mfn1 in GEN and FABP4 which mediated inflammation and lipid accumulation were explored. The knockdown efficiency of Mfn1 was verified in Figure 4A. Mfn1 SiRNA hampered the inhibitory effect of GEN on TNF-α and IL-6 (Figure 4B,C). Mfn1 SiRNA also abrogated the augment of Mfn1 by GEN in response to the LPS/HG/PA challenge (Figure 4D,E). The downregulated effect of GEN on bodipy fluorescence intensity was also prevented by Mfn1 SiRNA (Figure 4F). Moreover, the downregulated effect of BMS on IL-6 was blocked by Mfn1 SiRNA (Figure 4G,H). Mfn1 knockdown restrained the BMS-mediated inhibitory effect on FABP4 and promoted effect on Mfn1 (Figure 4I–K). The results suggest that Mfn1 was involved in the attenuated effect of GEN and BMS on inflammation and lipid accumulation.

### 3.6. GEN Targeted FABP4 to Inhibit Inflammation, Lipid Accumulation and Mitochondrial Fusion

**Figure 5:**
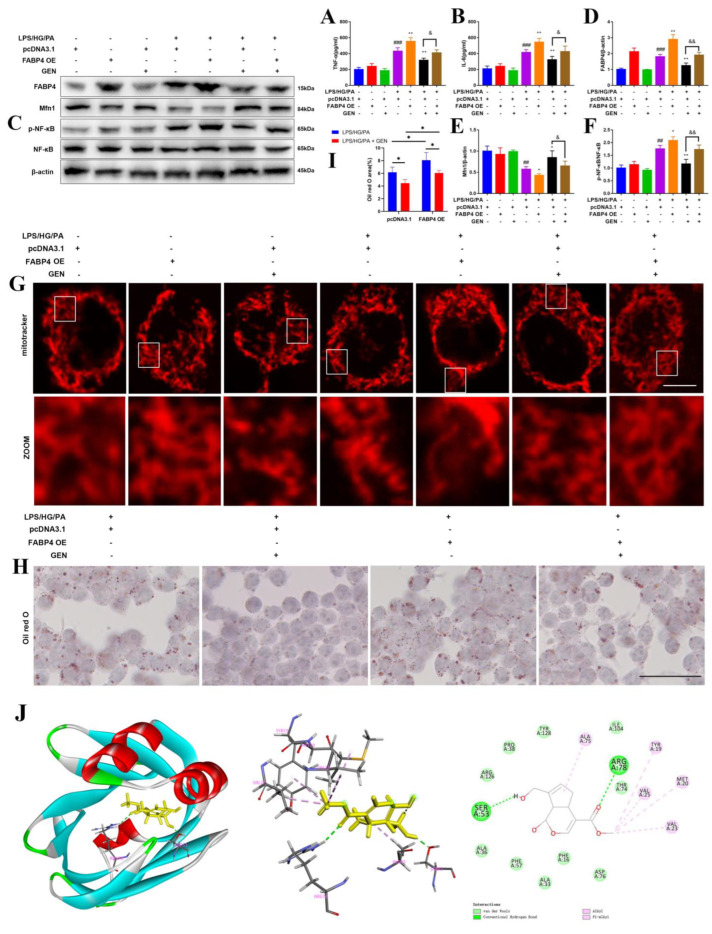
GEN targeted FABP4 to inhibit lipid accumulation and promote mitochondrial fusion in LPS/HG/PA-induced HMC3 cells. The HMC3 cells were treated with FABP4 overexpression plasmid (FABP4 OE) or pcDNA3.1 plasmid. The cells were treated with GEN for 4 h and then stimulated with LPS/HG/PA for 12 h. The supernatant concentrations of TNF-α and IL-6 were examined by ELISA (**A**,**B**) (n = 4). The protein expressions of FABP4, Mfn1, p-NF-κB and NF-κB were detected by Western blot (**C**–**F**) (n = 3). The mitochondrial morphology was observed by immunofluorescence staining. The scale bar equaled 5 μm (**G**). The lipid accumulation was visualized by oil red O staining. The scale bar equaled 50 μm (**H**). The analysis of oil red O staining was presented (**I**). The molecular docking was conducted by Discovery Studio (**J**). The results are expressed as means ± SDs. ^##^ *p* < 0.01, ^###^ *p* < 0.001 compared with control group. * *p* < 0.05, ** *p* < 0.01 compared with LPS/HG/PA group or the other group. ^&^ *p* < 0.05, ^&&^ *p* < 0.01 compared with LPS/HG/PA + pcDNA3.1 + GEN group.

The FABP4 overexpression plasmid was transfected into HMC3 cells to further investigate the mechanism. FABP4 overexpression increased the supernatant contents of TNF-α and IL-6 compared with those of the LPS/HG/PA group. The inhibitory activities of GEN on inflammatory cytokines were blocked by FABP4 overexpression (Figure 5A,B). The transfection with the FABP4 plasmid suppressed Mfn1 expression and enhanced NF-κB phosphorylation. The GEN-mediated upregulation of Mfn1 and the downregulation of p-NF-κB were hampered by the co-transfection with the FAPB4 plasmid (Figure 5C–F). The overexpression of FABP4 conduced to more severe mitochondrial fission as compared to that in the LPS/HG/PA group. The GEN-augmented mitochondrial fusion was abrogated by FABP4 overexpression (Figure 5G). The overexpression of FABP4 accelerated lipid accumulation. GEN treatment reduced lipid accumulation, which was blocked by the co-treatment with the FABP4 overexpression plasmid (Figure 5H,I). The data displayed that FABP4 was involved in GEN-attenuated inflammation, lipid accumulation and mitochondrial fusion in LPS/HG/PA-induced HMC3 cells. The molecular docking illustrated that the interactions between FABP4 and GEN included van der Waals interaction, conventional hydrogen bond, alkyl and Pi-alkyl interaction. GEN interacted with FABP4 by hydrogen bond at the SER53 and ARG78 residues. PHE16, ALA33, ALA36, PRO38, PHE57, THR74, ASP76, ILE104, ARG126 and TYR128 formed van der Waals interaction with GEN. TYR19, MET20, VAL23, VAL25 and ALA75 formed alkyl and Pi-alkyl interactions with GEN. The ARG78 aminonacyl-GEN may enhance the binding between FABP4 and GEN, which increases the inhibitory effect of GEN on FABP4. The CDOCKER interaction energy was −30.0787 kcal/mol (Figure 5J). As suggested in InterPro, there exited cytosolic fatty-acid binding domain (CFABD) in FABP4. The three CFABDs of FABP4 that interacted with GEN were shown in Figure S6. Our data showed that GEN may combine with FABP4. The effect of GEN-induced microglia polarization in diabetic cognitive impairment may be due to the mediation of FABP4.

### 3.7. GEN Promoted Phagocytosis of HMC3 Cells in Response to LPS/HG/PA Stimulation

Phagocytosis, the key feature of microglia, is responsible for scavenging debris and immune stimuli to maintain the homeostasis of the central nervous system. The effect of GEN on phagocytosis was assessed by the mRNA expressions of Triggering Receptor Expressed On Myeloid Cells 2 (*TREM2*), Macrophage Scavenger Receptor 1 (*MSR1*) and Scavenger Receptor Class B Member 1 (*SCARB1*). GEN promoted the transcription of *TREM2* and slightly enhanced the transcription of *MSR1* (Figure S5A–C). The immunofluorescence staining using Dextran showed that GEN increased the phagocytosis activity of microglia (Figure S5D,E). Our result implies that GEN promoted phagocytosis in LPS/HG/PA-induced HMC3 cells.

### 3.8. GEN Inhibited Mfn1 Ubiquitination via Hrd1

**Figure 6:**
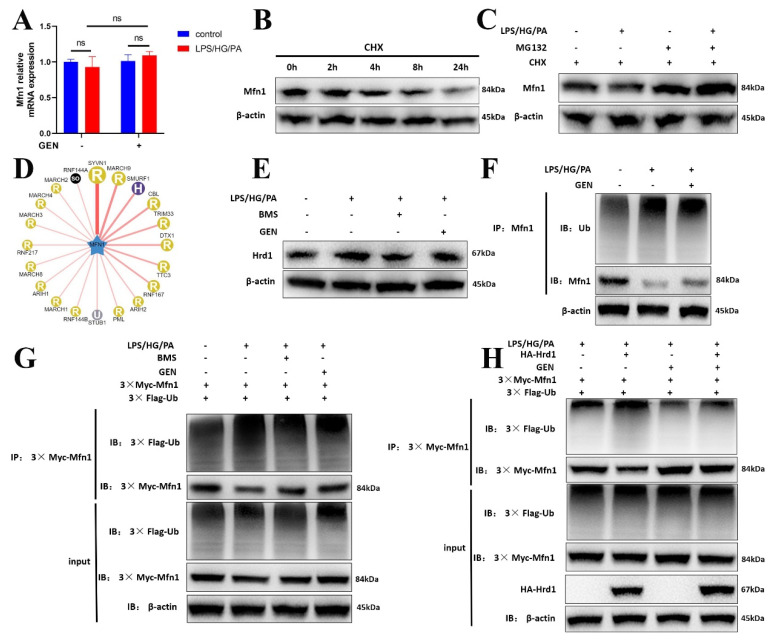
The effect of GEN on the ubiquitination of Mfn1. The HMC3 cells were treated with GEN (20 μM) for 4 h and then stimulated with LPS/HG/PA for 12 h. The *MFN1* mRNA expression was measured by PCR (**A**). HMC3 cells were treated with CHX (10 μg/mL) and the proteins were extracted at various times (**B**). HMC3 cells were exposed to LPS/HG/PA stimulation, CHX (10 μg/mL) and MG132 (10 μM). The protein expression of Mfn1 in HMC3 cells was detected by Western blot (**C**). The E3 ligase was predicted by ubibrowser (**D**). HMC3 cells treated with GEN (20 μM) or BMS for 4 h and then stimulated with LPS/HG/PA for 12 h. The Hrd1 protein expression was measured by Western blot (**E**). HMC3 cells were treated with GEN (20 μM) for 4 h prior to LPS/HG/PA stimulation. The ubiquitination of Mfn1 was detected by co-IP (**F**). 293T cells were transfected with 3× Myc-Mfn1 and 3× Flag-Ub plasmids prior to the treatment with GEN (20 μM) or BMS. After 4 h, the cells were exposed to LPS/HG/PA for 12 h. The ubiquitination of 3× Myc-Mfn1 was detected by co-IP (**G**). 293T cells were transfected with 3× Myc-Mfn1, 3× Flag-Ub and HA-Hrd1 plasmids prior to the treatment with GEN (20 μM). After 4 h, the cells were exposed to LPS/HG/PA for 12 h. The ubiquitination of 3× Myc-Mfn1 was detected by co-IP (**H**). The results are expressed as means ± SDs. ns means not significant.

Our experiment found that GEN upregulated Mfn1 expression. To further explore its potential mechanism, the *Mfn1* mRNA expression was assessed. It was found that GEN treatment did not significantly alter Mfn1 transcription (Figure 6A). We assumed that the modulated effect of GEN on Mfn1 may be due to its post-translational modification. The protein degradation was detected by CHX and it was shown that Mfn1 was degraded (Figure 6B). With the co-treatment with proteasome inhibitor MG132, we found that the proteolysis of Mfn1 may be attributed to ubiquitination (Figure 6C). We predicted the E3 ubiquitin ligase and found that Hrd1, namely Synoviolin 1 (SYVN1), may be the critical ligase (Figure 6D). GEN and BMS inhibited the expression of Hrd1 (Figure 6E). The treatment with GEN inhibited the ubiquitination of Mfn1 in LPS/HG/PA-induced HMC3 cells (Figure 6F). GEN and BMS treatments restrained the ubiquitination of 3× Myc-Mfn1 in 293T cells (Figure 6G). Herein, the HA-Hrd1 plasmid was also transfected to 293T cells. It was found that HA-Hrd1 enhanced the ubiquitination of Mfn1 in LPS/HG/PA-exposed 293T cells, which was blocked by GEN treatment (Figure 6H). The data implied that GEN inhibited Mfn1 ubiquitination, which was mediated by Hrd1.

### 3.9. GEN Attenuated Diabetic Cognitive Impairment by Inhibiting Microglia Inflammation

**Figure 7:**
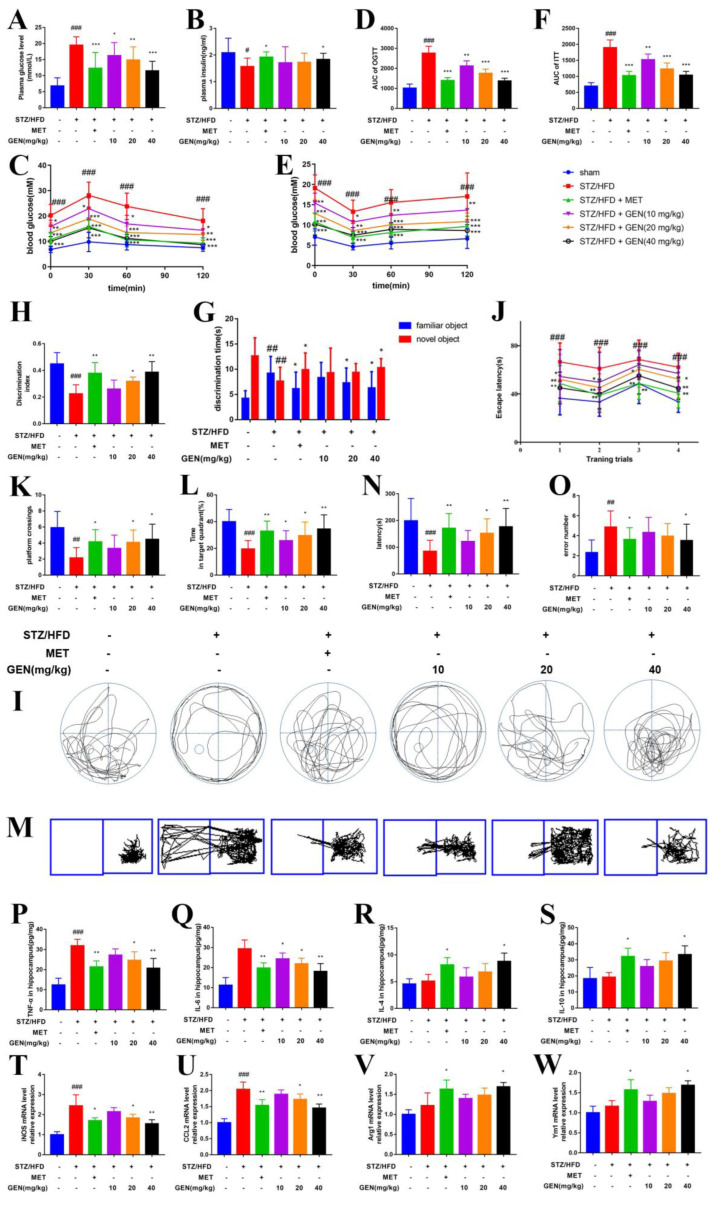
GEN attenuated diabetic cognitive impairment by mediating microglia polarization indicators. The mice were fed with HFD and intraperitoneally injected with STZ. Then, GEN (10, 20, 40 mg/kg) treatment was applied. The plasma glucose (**A**) and insulin (**B**) levels were measured. The OGTT (**C**) and ITT (**E**) were conducted. The AUCs of OGTT (**D**) and ITT (**F**) were calculated. The discrimination time (**G**) was monitored and the discrimination index (**H**) was calculated in novel object recognition. The representative swimming track (**I**), escape latency on day 1–4 (**J**), platform crossing times (**K**) and time in target quadrant (%) (**L**) were recorded in Morris water maze. The representative moving track (**M**), latency (**N**) and the error number (**O**) were monitored in passive avoidance test (n = 15–16). The concentrations of TNF-α (**P**), IL-6 (**Q**), IL-4 (**R**) and IL-10 (**S**) in hippocampi were determined by ELISA (n = 4). The mRNA expressions of *iNOS* (**T**), *Ccl2* (**U**), *Arg1* (**V**) and *Ym1* (**W**) in hippocampi were measured by PCR (n = 4). The results are expressed as means ± SDs. ^#^ *p* < 0.05, ^##^ *p* < 0.01, ^###^ *p* < 0.001 compared with sham group. * *p* < 0.05, ** *p* < 0.01, *** *p* < 0.001 compared with STZ/HFD group.

**Figure 8:**
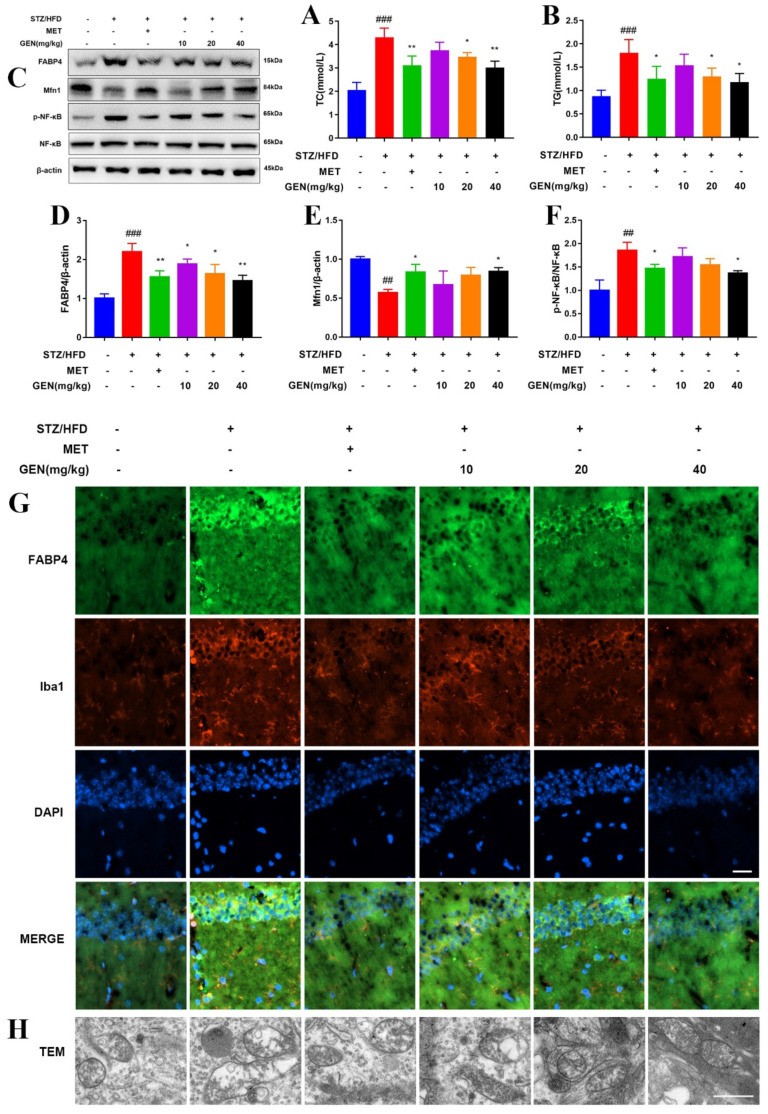
GEN attenuated diabetic cognitive impairment by inhibiting lipid accumulation and promoting mitochondrial fusion via FABP4/Mfn1. The mice were fed with HFD and intraperitoneally injected with STZ. Then, GEN (10, 20, 40 mg/kg) treatment was applied. The levels of TC (**A**) and TG (**B**) in serum were determined (n = 4). The hippocampi protein expressions of FABP4, Mfn1, p-NF-κB and NF-κB were detected by Western blot (**C**–**F**) (n = 3). The expressions of FABP4 and Iba1 were observed under immunofluorescence microscope. The scale bar equaled 50 μm (**G**). The mitochondrial morphology was visualized by TEM (**H**). The results are expressed as means ± SDs. ^##^ *p* < 0.01, ^###^ *p* < 0.001 compared with sham group. * *p* < 0.05, ** *p* < 0.01 compared with STZ/HFD group.

The in vivo experiment employed the STZ/HFD-induced diabetic cognitive impairment murine model. The present study was carried out with three dosages of GEN (10 mg/kg, 20 mg/kg, 40 mg/kg) and MET. GEN and MET administrations effectively reduced the plasma glucose concentration in STZ/HFD-induced mice (Figure 7A). GEN (40 mg/kg) and MET increased the insulin content (Figure 7B). GEN administration also attenuated glucose and insulin tolerance, as well as reduced the AUCs in OGTT and ITT (Figure 7C–F). The data implied that GEN reduced the symptoms of diabetes. GEN also increased the discrimination time to novel objects and decreased the discrimination time to familiar objects in novel object recognition, which was reflected by the increased discrimination index (Figure 7G,H), decreased escape latency, increased platform crossing times and time in target quadrant in Morris water maze (Figure 7I–L), as well as increased latency and reduced error number in passive avoidance test of diabetic mice (Figure 7M–O). The above results suggested that the diabetic cognitive impairment murine model was successfully established, and GEN treatment could attenuate diabetic cognitive impairment. GEN reduced the hippocampal levels of TNF-α, IL-6 and *iNOS*, *Ccl2* mRNA in STZ/HFD-induced mice in dose-dependent manners (Figure 7P,Q,T,U). GEN augmented the expressions of hippocampal IL-4 and IL-10 and the transcriptions of *Ym1* and *Arg1* (Figure 7R,S,V,W). The microglia were activated by STZ/HFD stimulation, which was inhibited by GEN treatment (Figure 8G). It was suggested that the GEN treatment suppressed M1 microglia polarization and promoted M2 microglia polarization. The efficacies of GEN on M1 phenotype biomarkers were better than those on M2 phenotype biomarkers. Thus, it was assumed that the attenuated effect of GEN on diabetic cognitive impairment may be attributed to the mediation of microglia inflammation.

### 3.10. GEN Decreased Lipid Accumulation and Promoted Mitochondrial Fusion via FABP4/Mfn1 in Hippocampi of STZ/HFD-Induced Mice

The serum contents of TC and TG were determined. As depicted in Figure 8A,B, GEN administration reduced the lipid concentration. GEN treatment also decreased the expressions of FABP4, p-NF-κB and upregulated Mfn1 expression (Figure 8C–F). The immunofluorescence staining of hippocampi with FABP4 and Iba1 was observed. The enhanced FABP4 expression in STZ/HFD-induced hippocampus was restrained by GEN administration (Figure 8G). FABP4 presented co-location with Iba1. TEM observation showed more mitochondrial fission in STZ/HFD-challenged mice as compared with those of the control group. GEN treatment contributed to less mitochondrial fission but more fusion (Figure 8H). Our research displayed that GEN reduced lipid accumulation and enhanced mitochondrial fusion in hippocampi via FABP4/Mfn1/NF-κB signaling.

## 4. Discussion

It is widely acknowledged that diabetic encephalopathy is induced by the combination of chronic HFD and STZ. STZ, the nitrosamine compound, is commonly peripherally injected to induce experimental diabetic animals. It was widely acknowledged that the low-dosage injection of STZ (less than 65 mg/kg) for two or three times accompanied with HFD was the classical method to induce T2DM. Hyperglycemia and hyperlipidemia are also believed to be the initiators in diabetic encephalopathy development by inducing glucose metabolism/lipid metabolism disorder, neuronal damage, oxidative stress and inflammatory reaction. The present research confirmed that GEN ameliorated diabetic cognitive impairment by reducing blood glucose levels and improving abnormal cognitive behavior.

The excessive ROS generated in hyperglycemia or hyperlipidemia conditions contributes to antioxidant deficiency, brain morphological abnormality and cognitive impairment. ROS is identified as the etiological factor in the development of Alzheimer’s disease, diabetes and diabetic cognitive impairment. NOX is the enzyme transferring electrons through biological membranes. As the main source of ROS, the NOX system is triggered by various factors to produce ROS via the translocation of p47phox and p67phox to the membrane. The localization of p47phox to the membrane further brings p67phox into contact with NOX2, which is the pivotal step for NOX2 activation and complex assembly. Then, superoxide is generated by transferring electrons from NADPH to electron acceptor oxygen which is located in luminal or extracellular space. As the prototype of NOX, NOX2 (gp91phox) has been investigated in phagocytes. Once triggered by phagocyte stimuli, NOX2 fuses with the phagosomal or plasma membrane to modulate immune response [23,24]. Enhanced NOX2 signaling elevated the level of intracellular ROS and triggered inflammatory cascade in macrophage and microglia. It was illustrated that gp91phox knockout prevented microglia activation and that NOX2 inhibitor attenuated cognition in sepsis-related cognitive impairment [25]. Upon the inflammatory stimulation, microglia activates transcription factor nuclear factor-kappa B (NF-κB). NF-κB phosphorylates and translocates into the nucleus to govern the transcription of various inflammatory mediators including NOX2. The overproduced intracellular ROS mediated by NOX2 further activates NF-κB, which contributes to persistence inflammation [26]. We found that the inflammation as well as high glucose and high fatty acid in diabetes increased ROS generation and caused mitochondrial dysfunction, which were restored by GEN and BMS interventions. The inhibitory effect of GEN on ROS was due to the inhibition of NOX2 and p47phox/p67phox membrane translocation. The application of the ROS inhibitor NAC proved that ROS was involved in GEN-mediated inflammation and lipid accumulation.

Neuroinflammation is the cellular reaction of the central nervous system upon infection and lesion. It is featured by the excessive generation of chemokine, inflammatory cytokine and ROS, which deteriorate the pathology of diabetic cognitive impairment. Microglia are a vital source of oxidative stress factor, nitric oxide, neurotoxic substance, tumor necrosis factor and even interleukin. Microglia are kept in resting state during physiological condition. Once activated by inflammatory stimuli, microglia participate in the immune regulation by switching to M1 or M2 polarization and secreting different biomarkers. Hyperglycemia/hyperlipidemia disturb the M1/M2 balance and shift toward pro-inflammatory M1 phenotype. The robust upregulations of TNF-α and IL-6 further aggravate high glucose/high fatty acid-caused central lesion. The current work depicted that the GEN and FABP4 inhibitors prevented M1 microglia polarization.

As highly mobile organelle, mitochondria impart multiple effects including energy provision, inflammation and lipid oxidation, which are tightly related to mitochondrial dynamics. Mitochondrial dynamics modulate T cells differentiation and macrophage polarization. Mitochondrial fusion occurs in OMM and in the inner membrane of mitochondria (IMM). OMM fusion is mediated by Mfn1 and Mfn2 [27]. The suppression of Mfn1 caused fragmented mitochondria and inhibited mitochondria membrane fusion. The fatty acid did not distribute homogenously in the mitochondria of the Mfn1 deficiency cell, which caused high or low concentrations of fatty acid in individual mitochondrial elements. Mfn1 knockout cells stored more fatty acid than wild-type cells [28]. The lipid metabolism was blocked in Mfn1^−/−^ alveolar type 2 epithelial cells [29]. The blockade of mitochondrial fusion via Mfn1 inhibition was closely associated with the process of insulin resistance and obesity [30]. The mitochondrial ROS was augmented by the overexpression of Drp1, while inhibited by the knockdown of Drp1 [31]. Our results display that GEN promoted Mfn1 and mitochondrial fusion, which was accompanied by the reductions in inflammatory cytokines and lipid accumulation.

Post-translational modifications (PTMs) are reversible biological progressions which enable the adaptation to multiple intracellular and extracellular alterations. Ubiquitination, one of the important PTMs, is the covalent fusion process of ubiquitin to target protein on various lysine (K) residues. The ubiquitin-linked protein is degraded by the ubiquitin proteasome system, which mediates the expression of target proteins. E3 ubiquitin ligase is the critical enzyme deciding the substrate specificity [32]. Bioinformatic analysis predicted that Hrd1 may be the critical E3 ubiquitin ligase for Mfn1. To further explore the mechanism by which GEN upregulated Mfn1, we performed co-IP. As expected, GEN inhibited Mfn1 ubiquitination and Hrd1 may be the E3 ligase of Mfn1.

As the receptor for fatty acids, fatty acid-binding proteins (FABPs) control metabolism, lipid trafficking, immune and inflammatory reactions. The reversible combination of FA including PA and FABP4 regulates a variety of lipid-associated processes, including mitochondria dysfunction, oxidative stress and inflammation. Long-chain fatty acids (LCFA), including PA, alter FABP4 activity. FABP4 mainly distributes in adipocyte and macrophage, which implies that FABP4 may be a target for diabetic-related inflammation. Diminished adiposity and improved insulin sensitivity were found in FABP4-Cre mice [33]. Stable, high glucose (30 mM) for 24 h increased the protein expression and transcription of FABP4 via the binding between the FABP4 promoter and highly conserved AP-1 cis-element [34]. Free fatty acid was regarded as the agonist of Toll Like Receptor (TLR), which induced inflammatory cascade in macrophage of T2DM patients [35]. A serum FABP4 level was identified as the prognostic molecule in T2DM and stroke patients [36]. The treatment with the FABP4 inhibitor reduced TNF-α and iNOS expressions in microglia [14]. Growing evidence has emerged indicating that FABP4 inhibitors are effective treatment approaches for T2DM. BMS, a biphenyl azole compound, is the most commonly used FABP4 inhibitor. BMS suppresses fatty acid uptake, lipid accumulation, insulin resistance, oxidative stress and inflammation [37]. BMS treatment contributed to decreased blood glucose, improved glucose tolerance, and downregulated inflammatory signaling in ob/ob mice [38]. Thus, the inhibition of FABP4 could attenuate diabetic complication and neuro-inflammation. We assumed that FABP4 may be the target of microglia inflammation in diabetic cognitive impairment. Our results indicate that FABP4 inhibition suppressed microglia inflammation, ROS generation and lipid accumulation. Molecular docking also predicted the binding sites between GEN and FABP4.

**Figure 9:**
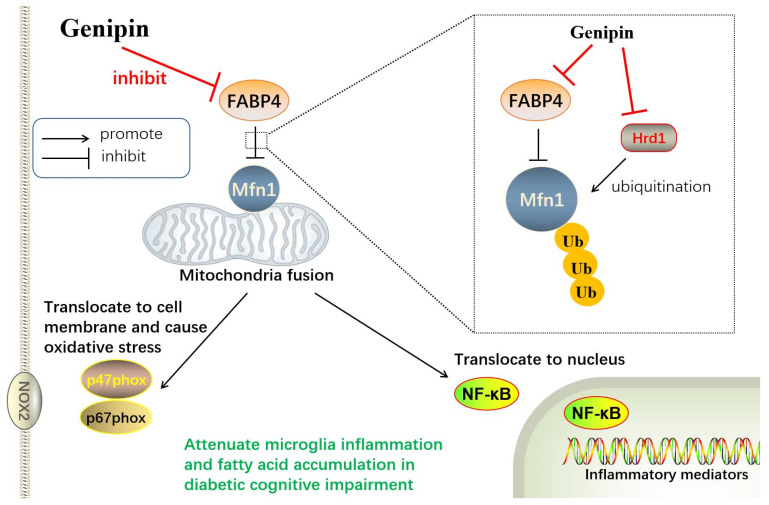
The potential mechanism of the present study.

We assessed the mRNA expressions of fatty acid β-oxidation, fatty acid uptake genes and fatty acid synthesis genes. The FABP family regulates fatty acids’ trans-membrane transportation and accelerates free fatty acid absorption. FABP4 forms a concentration gradient in the cell membrane. It was reported that FABP4 had an affinity to combine and deliver long-chain unsaturated fatty acids into the mitochondria [39]. Our research found that the fatty acid synthesis genes were altered limited in response to diabetic stimuli in microglia. We assumed that microglia were not the metabolism active cells; thus, the production of fatty acid was not the main function of microglia. The diabetic environment caused stress on microglia, such as contributing to the passive uptake and degradation of fatty acid, as well as mitochondrial fission and ROS overproduction, but not fatty acid synthesis. The potential mechanism was illustrated in Figure 9.

In conclusion, the present study found that GEN attenuated diabetic cognitive impairment by inhibiting microglia inflammation, lipid accumulation and promoting mitochondrial fusion, which was possibly regulated by FABP4 and Mfn1. Transgenic animals may be used in future investigations.

## Figures and Tables

**Table 1:** The primer sequence for PCR. H- means *Homo sapiens*, M- means *Mus musculus*.

Gene	Accession Number	Forward Primer (from 5′ to 3′)	Reverse Primer (from 5′ to 3′)
H-*iNOS*	NM_000625.4	AGGTCCAAATCTTGCCTGGG	TTGTTACCGCTTCCACCCTG
H-*CCL2*	NM_002982.4	CTCGCCTCCAGCATGAAAGT	GGTGACTGGGGCATTGATTG
H-*ARG1*	NM_000045.4	TTATGGGGACCTGCCCTTTG	CACCAGGCTGATTCTTCCGT
H-*YM1*	NM_001276.4	GATGTGACGCTCTACGGCAT	ACTCTGGGTGTTGGAGGCTA
H-*MFN1*	NM_033540.3	TTGCTGTTGCCGGGTGATAG	GCAGTAATCGCCTTCTTAGCC
H-*FABP3*	NM_001320996.2	AGCCTAGCCCAGCATCACTA	AGGGTAGGGGGAAGGTTATGA
H-*FABP4*	NM_001442.3	CCTTAGATGGGGGTGTCCTG	AACGTCCCTTGGCTTATGCT
H-*FABP5*	NM_001444.3	AGGAGTGGGAATAGCTTTGCG	TCTGCCATCAGCTGTGGTTTC
H-*FABP7*	NM_001319039.2	GCAGGTGGGAAATGTGACCA	GGCTAACAACAGACTTACAGTTTC
H*-ACOX1*	NM_001185039.2	GAGTTTGGCATCGCTGACCC	GAGTCCTTTGGATGAAAGCAGC
H*-ACAA2*	NM_006111.3	ATCGGGTGTAGCTGATGGTG	AGGGACAGGACCAATACCCA
H-*ECHS1*	NM_004092.4	GCTGCTGTCAATGGCTATGC	ACCAGTGAGGACCATCTCCA
H-*FASN*	NM_004104.5	GGATCACAGGGACAACCTGG	GGGAGATGAGGGGAGTTCCT
H-*ACLY*	NM_001096.3	ACTTCGGCAGAGACAGGTAGA	CCGATTCTGGATGGCTGAGG
H-*SLC27A1*	NM_198580.3	CTCTCTGCTTCCCCAGGATG	GCACAGAGAGACCGAAGAGG
H-*PPARα*	NM_001001928.4	CCCTGTCTGCTCTGTGGACT	CAGAGTGGGCTTTCCGTGTC
H-*TREM2*	NM_001271821.2	GATGCGGGTCTCTACCAGTG	CTCAGCCCTGGAGATGCTGT
H-*MSR1*	NM_001363744.1	TCTGCTGTTGAGACGTTGGG	CCCACTGCTCCATACTTGGT
H-*SCARB1*	NM_001082959.2	AGTCAGGGGTGTTTGAAGGC	AGAGAAACAAGGGGGCACTG
H-*β-actin*	NM_001101.5	GAGCACAGAGCCTCGCCTTT	GAGGCGTACAGGGATAGCAC
M-*iNOS*	NM_001313921.1	GCTCTAGTGAAGCAAAGCCCA	TCTCTCCACTGCCCCAGTTT
M-*Ccl2*	NM_011333.3	ACCTGCTGCTACTCATTCACC	ATTCCTTCTTGGGGTCAGCA
M-*Arg1*	NM_007482.3	AGCCAGGGACTGACTACCTT	TTGGGAGGAGAAGGCGTTTG
M-*Ym1*	NM_009892.3	AGAAGGGAGTTTCAAACCTGG	GTCTTGCTCATGTGTGTAAGTG
M-*β-actin*	NM_007393.5	TGAGCTGCGTTTTACACCCT	GCCTTCACCGTTCCAGTTTT

## Data Availability

Data is contained within the article.

## Supplementary Materials

The following supporting information can be downloaded at: https://www.mdpi.com/article/10.3390/antiox12010074/s1, Figure S1: The mRNA expressions of various FABPs; Figure S2: NAC blocked the attenuated effects of GEN on lipid accumulation and inflammation; Figure S3: BMS inhibited lipid accumulation and ROS content in LPS/HG/PA-induced HMC3 cells; Figure S4: The overexpression of Mfn1 inhibited inflammation and lipid accumulation in LPS/HG/PA-induced HMC3 cells; Figure S5: GEN promoted phagocytosis of LPS/HG/PA-induced HMC3 cells; Figure S6: The three cytosolic fatty-acid binding domains (CFABDs) of FABP4 were illustrated.
