# Supplementary material for: Genipin Attenuates Diabetic Cognitive Impairment by Reducing Lipid Accumulation and Promoting Mitochondrial Fusion via FABP4/Mfn1 Signaling in Microglia
Source: Antioxidants (Basel). 2022 Dec 29;12(1):74. doi: 10.3390/antiox12010074 (PMC9854533; doi:10.3390/antiox12010074)
Supplement: Supplementary file 1 [file antioxidants-12-00074-s001.zip › antioxidants-2105045-supplementary-refreshed-highlight.pdf]

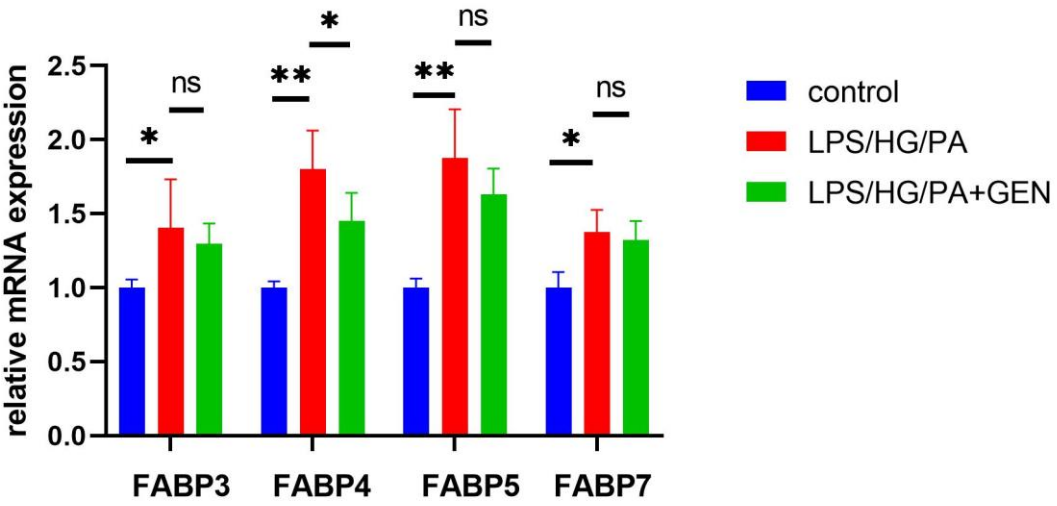

Fig S1. The mRNA expressions of various FABPs. The HMC3 cells were treated with GEN (20 μM) for 4 h and then stimulated with LPS/HG/PA for 12 h. The mRNA expressions of *FABP3*, *FABP4*, *FABP5*, *FABP7* in cells were assessed by PCR (n=4). The results were expressed as means ± SDs. *p < 0.05, **p < 0.01 compared with the other group.

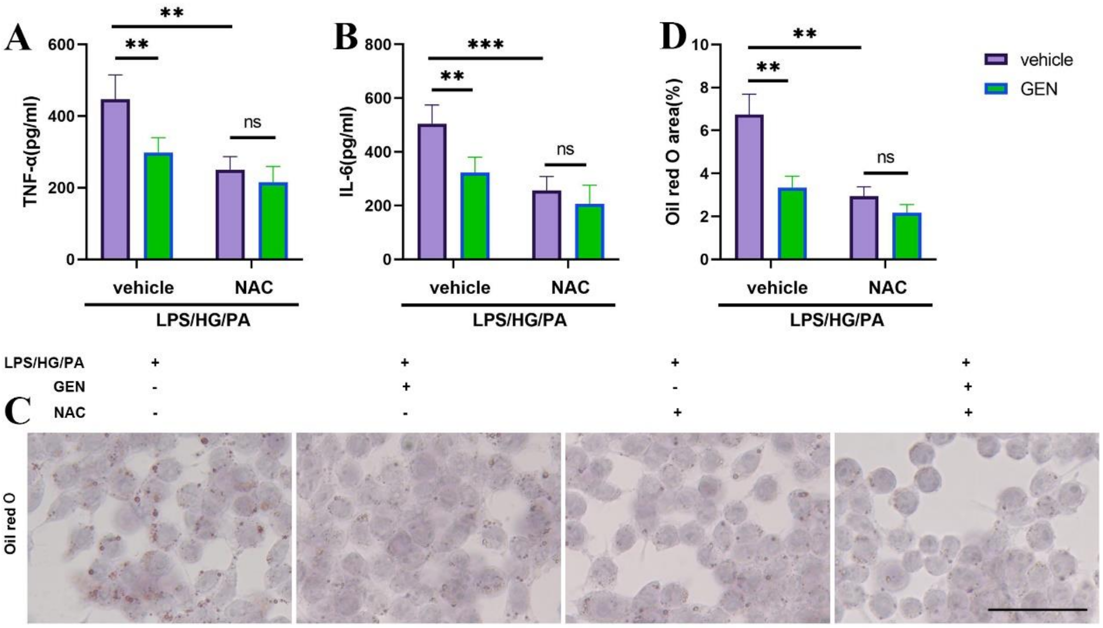

Fig S2. NAC blocked the attenuated effects of GEN on lipid accumulation and inflammation. The HMC3 cells were treated with NAC (1 mM) for 4 h and then stimulated with LPS/HG/PA for 12 h. The supernatant concentrations of TNF-*α* (A) and IL-6 (B) were examined by ELISA (n=4). The lipid accumulation was observed by oil red O staining. The scale bar equaled 50 μm (C). The analysis of

oil red O staining was presented (D). The results were expressed as means ± SDs. **p < 0.01, ***p < 0.001 compared with the other group.

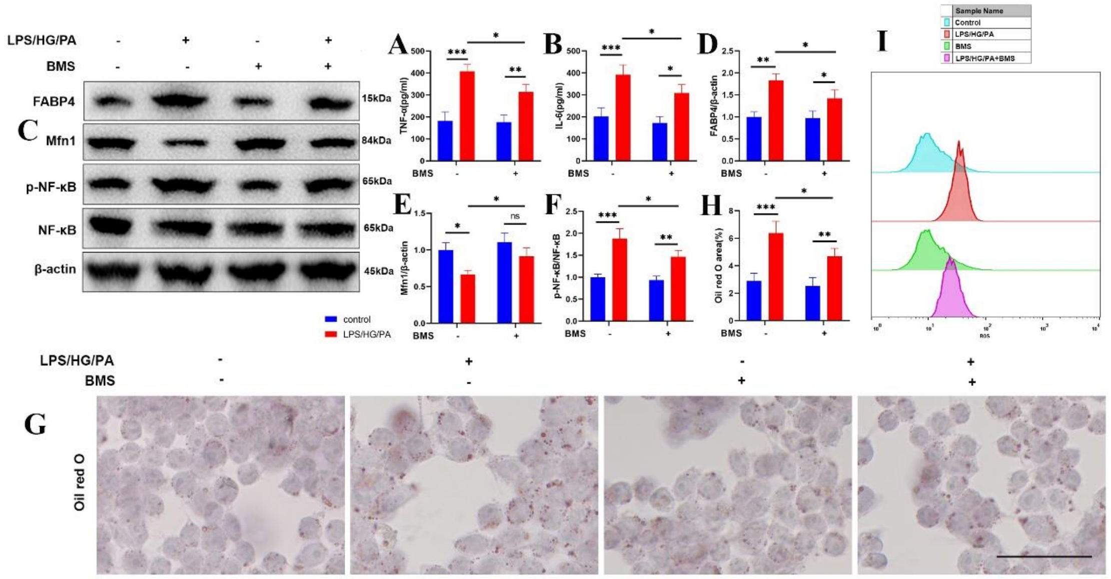

Fig S3. BMS inhibited lipid accumulation and ROS content in LPS/HG/PA-induced HMC3 cells. The HMC3 cells were treated with BMS (40 μM) for 4 h and then stimulated with LPS/HG/PA for 12 h. The supernatant concentrations of TNF-α (A) and IL-6 (B) were examined by ELISA (n=4). The protein expressions of FABP4, Mfn1, p-NF-κB and NF-κB were detected by western blot (C-F)(n=3). The lipid accumulation was observed by oil red O staining. The scale bar equaled 50 μm (G). The analysis of oil red O staining was presented (H). ROS positive cell counts were evaluated by flow cytometry (I). The results were expressed as means ± SDs. *p < 0.05, **p < 0.01, ***p < 0.001 compared with the other group.

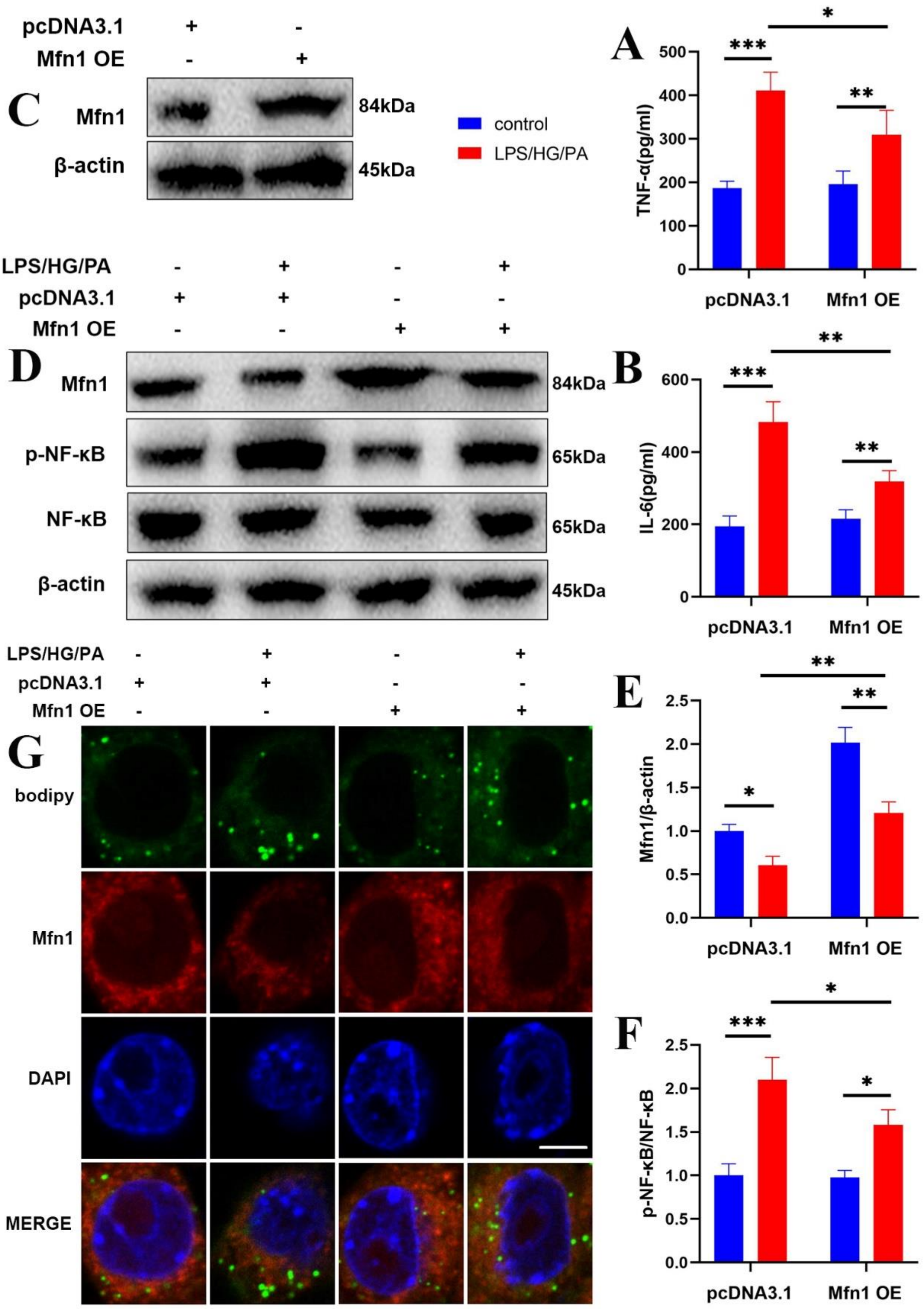

Fig S4. The overexpression of Mfn1 inhibited inflammation and lipid accumulation in LPS/HG/PA-induced HMC3 cells. The HMC3 cells were treated with Mfn1 overexpression plasmid (Mfn1 OE) or pcDNA3.1 plasmid (Mfn1 NC). The cells were then stimulated with LPS/HG/PA for 12 h. The supernatant concentrations of TNF-α (A) and IL-6 (B) were examined by ELISA (n=4). The

overexpression efficacy was verified by western blot (C). The protein expressions of Mfn1, p-NF-κB and NF-κB were detected by western blot (D-F)(n=3). The bodipy and Mfn1 were observed by immunofluorescence staining under laser confocal microscope. The scale bar equaled 5 μm (G). The results were expressed as means ± SDs. *$p < 0.05$, **$p < 0.01$, ***$p < 0.001$ compared with the other group.

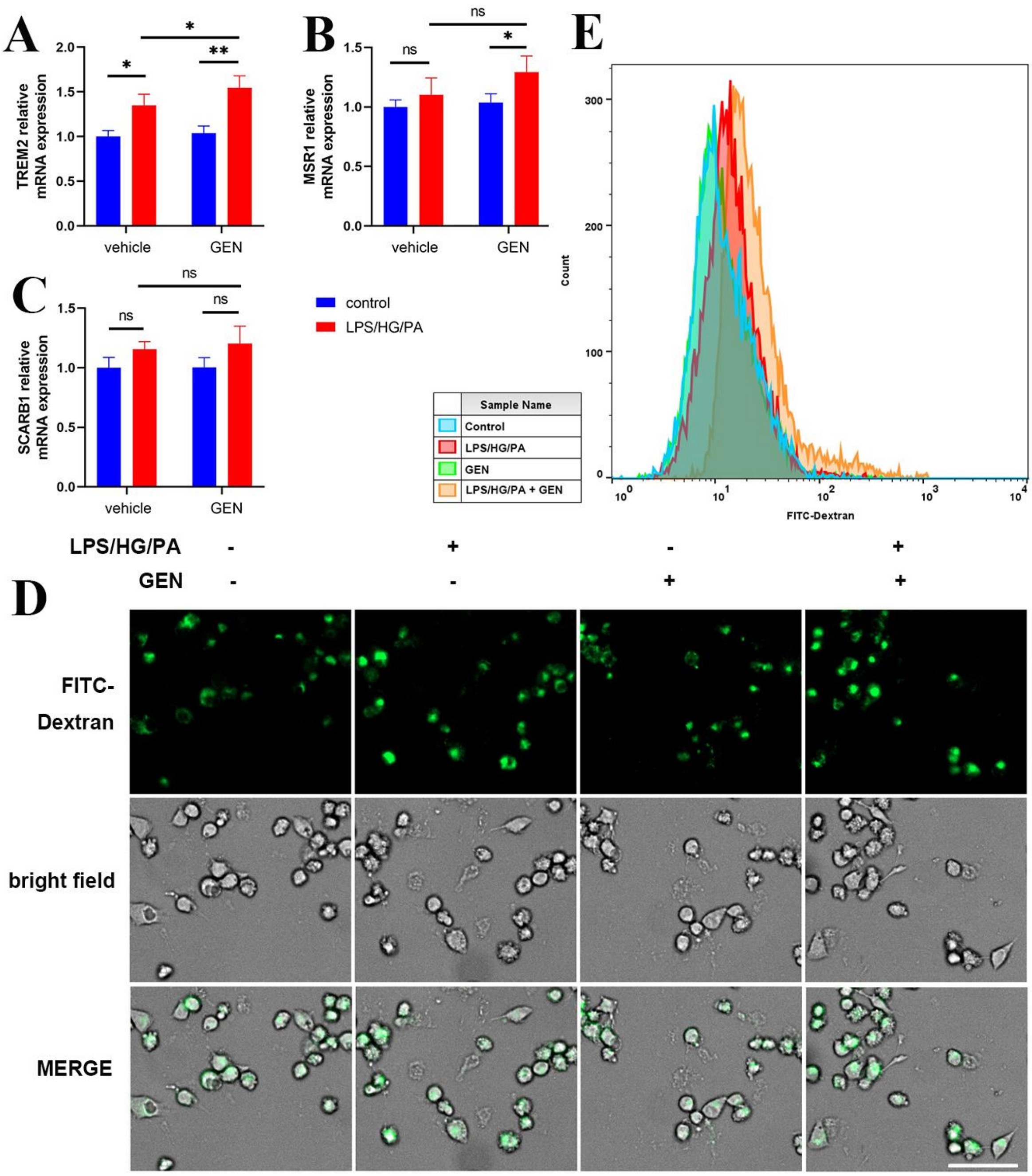

Fig S5. GEN promoted phagocytosis of LPS/HG/PA-induced HMC3 cells. The HMC3 cells were treated with GEN for 4 h. Then the cells were stimulated with GEN (20 μM) for 4 h prior to LPS/HG/PA stimulation for 12 h. The mRNA expressions of *TREM2* (A), *MSR1* (B), *SCARB1* (C) in cells were examined by PCR (n=4). The phagocytosis of microglia was evaluated using FITC-Dextran under fluorescence microscope. The scale bar equaled 50 μm (D). The phagocytosis was further investigated by flow cytometry(E). The results were expressed as means ± SDs. *p < 0.05, **p < 0.01 compared with the other group.

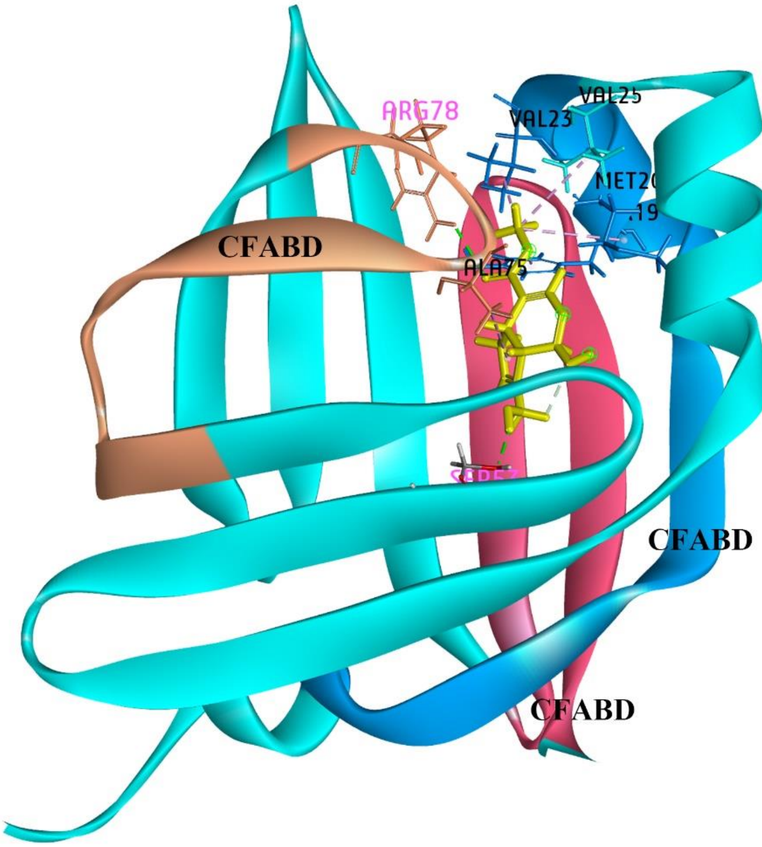

Fig S6. The three cytosolic fatty-acid binding domains (CFABDs) of FABP4 were illustrated. The sequences of these three domains were FVGTWKLVSSENFDDYMKEVGVG, SFILGQEFDEVTADDRK, and DDKLVVECVMKGVTSTRVYER.
